# Early stability and late random tumor progression of a HER2-positive primary breast cancer patient-derived xenograft

**DOI:** 10.1038/s41598-021-81085-y

**Published:** 2021-01-15

**Authors:** Lorena Landuzzi, Arianna Palladini, Claudio Ceccarelli, Sofia Asioli, Giordano Nicoletti, Veronica Giusti, Francesca Ruzzi, Marianna L. Ianzano, Laura Scalambra, Roberta Laranga, Tania Balboni, Maddalena Arigoni, Martina Olivero, Raffaele A. Calogero, Carla De Giovanni, Massimiliano Dall’Ora, Enrico Di Oto, Donatella Santini, Maria Pia Foschini, Maria Cristina Cucchi, Simone Zanotti, Mario Taffurelli, Patrizia Nanni, Pier-Luigi Lollini

**Affiliations:** 1 Laboratory of Experimental Oncology, IRCCS Istituto Ortopedico Rizzoli, Bologna, Italy; 2 Laboratory of Immunology and Biology of Metastasis, Department of Experimental, Diagnostic and Specialty Medicine (DIMES), University of Bologna, Viale Filopanti 22, 40126 Bologna, Italy; 3 Laboratory of Oncologic Immunocytopathology, DIMES, St. Orsola-Malpighi Hospital, University of Bologna, Via Massarenti 9, 40138 Bologna, Italy; 4 Department of Biomedical and Neuromotor Sciences, University of Bologna, Unit of Anatomic Pathology “M. Malpighi”, Bellaria Hospital, Via Altura, 3, 40139 Bologna, Italy; 5 Department of Molecular Biotechnology and Health Science, University of Torino, Turin, Italy; 6 Department of Oncology, University of Torino, Turin, Italy; 7 Candiolo Cancer Institute-FPO, IRCCS, Candiolo, 10060 Turin, Italy; 8 Pathology Unit, St. Orsola-Malpighi Hospital, University of Bologna, via Massarenti 9, 40138 Bologna, Italy; 9 Unit of Breast Surgery, Bellaria Hospital, AUSL Bologna, Bologna, Italy; 10 Department of Medical and Surgical Science, Bologna University-Breast Unit Sant’Orsola Hospital, Bologna, Italy

**Keywords:** Breast cancer, Cancer models, Cancer stem cells, Cancer therapy, Tumour heterogeneity

## Abstract

We established patient-derived xenografts (PDX) from human primary breast cancers and studied whether stability or progressive events occurred during long-term in vivo passages (up to 4 years) in severely immunodeficient mice. While most PDX showed stable biomarker expression and growth phenotype, a HER2-positive PDX (PDX-BRB4) originated a subline (out of 6 studied in parallel) that progressively acquired a significantly increased tumor growth rate, resistance to cell senescence of in vitro cultures, increased stem cell marker expression and high lung metastatic ability, along with a strong decrease of BCL2 expression. RNAseq analysis of the progressed subline showed that BCL2 was connected to three main hub genes also down-regulated (CDKN2A, STAT5A and WT1). Gene expression of progressed subline suggested a partial epithelial-to-mesenchymal transition. PDX-BRB4 with its progressed subline is a preclinical model mirroring the clinical paradox of high level-BCL2 as a good prognostic factor in breast cancer. Sequential in vivo passages of PDX-BRB4 chronically treated with trastuzumab developed progressive loss of sensitivity to trastuzumab while HER2 expression and sensitivity to the pan-HER tyrosine kinase inhibitor neratinib were maintained. Long-term PDX studies, even though demanding, can originate new preclinical models, suitable to investigate the mechanisms of breast cancer progression and new therapeutic approaches.

## Introduction

Patient-derived tumor xenografts (PDX) are candidate to successfully replace cell-based tumor xenografts in many fields of experimental oncology, from the search of new early biomarkers or targets, to the development and testing of novel drugs^1–4^. PDX, compared to cell-derived xenografts (frequently of clonal origin and artificially selected for in vitro growth), better reproduced patient tumor populations complexity, molecular inter- and intra-tumor heterogeneity, dynamic tumor plasticity and microenvironmental interactions^5,6^, even if with some limitations mainly concerning immune interactions^7^. On the whole, PDX and other cancer models, such as murine and human cancer cell lines and organoids or in silico models^8,9^, are not alternative but complementary systems to obtain a better understanding of cancer biology.

PDX have been obtained with a relatively high rate of engraftment from many tumor types, usually displaying high grade of malignancy at the time of surgery, such as colorectal, ovarian and lung carcinomas, melanoma and bone sarcomas^2,10,11^. On the contrary, establishing PDX from breast cancer (like from other hormone-related tumors such as prostatic tumors) was rather difficult. Breast cancer PDX usually showed very long latency and slow growth rate, even when using severe immunodeficient mice, orthotopic engraftment and hormone supplement^2^. In breast cancer, the percentage of established PDX in serial passages drops down to 10–20%^3,12,13^. The rate of successful PDX depends on the frequency of high-grade and high-proliferating tumors in the cohort of patients studied^14^. Adaptation to in vivo growth selects for the most aggressive tumors, often with a correlation with bad prognosis in the patient^6,14,15^, and most available breast cancer PDX models belong to the highly malignant triple-negative subtype^3^.

Breast cancers are dynamic entities that undergo variation, selection and progression within the patient. To investigate whether PDX models recapitulate such tumor dynamics along time, we established primary breast cancer PDX, representative of the main subtypes, and studied whether functional stability or progressive events occurred to human breast cancers passaged in vivo up to some years.

## Methods

### Clinical series

Fresh primary breast cancer specimens from 61 previously untreated non-consecutive patients of the S. Orsola-Malpighi Hospital, Bologna and of the Bellaria Hospital, Bologna were received by the animal facility after surgical resection and immediately implanted orthotopically in the mammary fat pad of immunodeficient mice (see Supplementary Table S1). As the projects approved by Ethical Committees did not include the follow-up of patients, and mandated data anonymization, no further data is available to us regarding subsequent treatments or patients’ fate.

### Mice

5–10 weeks-old, NOD-SCID-Il2rg^−/−^ (NSG) female mice (breeders received from Jackson Laboratories) or BALB/cRag2^−/−^Il2rg^−/−^ (BRG) female mice (breeders received from Drs T. Nomura and M. Ito, Central Institute for Experimental Animals, CIEA, Kawasaki, Japan) were kept under sterile conditions and were used for human tumor fragment implantation and propagation. A few clinical specimens (BRB4, BRS1, BRS2, BRS7, BRS9, BRS13, BRS25) were implanted in BRG mice, all other specimens were implanted in NSG mice. A direct comparison of PDX implantation in NSG or BRG mice did not reveal any significant difference in tumor growth (data not shown).

### PDX establishment and propagation

Fresh fragments of human primary breast tumors were aseptically collected during surgical procedures at S. Orsola-Malpighi Hospital, Bologna or at Bellaria Hospital, Bologna, put in cold Dulbecco’s modified Eagle medium supplemented with 10% Fetal Bovine Serum (FBS) (Thermo Fisher Scientific, USA), stored on ice and immediately delivered to the animal facility. Tumor fragments, with a diameter of 3–4 mm, were implanted in the fourth left mammary intact fat pad of anesthetized female mice. Mice were inspected weekly for tumor growth. Tumor diameter was measured with sterile calipers and volume was calculated using the formula π[√(*a* × *b*)]^3^/6 where *a* = maximal tumor diameter and *b* = major tumor diameter perpendicular to *a*. Masses > 10 mm^3^ were scored as tumors. When tumors reached a volume of 1.5–1.9 cm^3^, animals were euthanized by CO_2_ inhalation and an accurate necropsy was performed. Tumors were resected and divided into representative samples to be processed for the following applications: propagation by serial in vivo passage (as above); histology, immunohistochemistry and fluorescent in situ hybridization (FISH) analysis; establishment of cell cultures; immunofluorescence and cytofluorometric analysis; directly frozen in vials, in liquid nitrogen, for nucleic acids or protein extraction. Organs such as ovaries, lungs, brain and bone marrow were processed for the detection of metastatic dissemination. For the detailed procedures, see Supplementary Methods S1. Aliquots of tumor fragments were stored frozen in 90% FBS–10% dimethyl sulfoxide.

### Metastatic ability

Tumor fragments were dissociated by incubation in 0.05% Trypsin–0.002% EDTA at 37 °C for 5 min and passed through a 70 µm cell strainer (Becton Dickinson, Bedford, MA, USA) to obtain single-cell suspension. Cell number and viability were determined by vital counting in a hemocytometer. Cells were administered intravenously (i.v.) in a tail vein at doses ranging from 0.5 to 2 × 10^6^ cells in 0.4 ml Phosphate-Buffered Saline (PBS). Mice were inspected weekly and euthanized as above at any initial sign of metastatic growth or after 40–70 weeks. An accurate necropsy was performed: lungs, brain, ovaries and femoral bone marrow were collected for molecular detection of metastatic dissemination (see Supplementary Methods S1) or, in case of overt lung colonization, metastases were counted at a dissection microscope.

### Drug treatment

Mice entered drug treatment when PDX reached a volume of 10 mm^3^ and were randomly assigned to control group or to drug treatment (see Statistics). Trastuzumab (Herceptin, Roche) was given lifetime twice per week at a 4 mg/kg dose, diluted in saline, through intraperitoneal (i.p.) injection. Neratinib (kindly provided by Puma Biotechnology, Inc., Los Angeles, CA, USA) was administered by oral gavage at a 40 mg/kg dose^16,17^, dissolved in 0.5% methylcellulose–0.4% tween 80, 5 times per week, for at least 13–15 weeks. Tamoxifen or 17β-estradiol (Innovative Research of America, Sarasota, FL, USA) were administered by subcutaneous implantation of a pellet releasing respectively 0.5 mg of tamoxifen over 60 days or 0.72 mg of 17β-estradiol over 90 days. Control groups did not receive any treatment. To investigate mechanisms of action of the different drugs in vivo, mice bearing 1 cm^3^ breast cancer PDX were treated for 4 consecutive days with trastuzumab alone (4 mg/kg given i.p.), with tamoxifen alone (pellet as above), with neratinib alone (40 mg/kg, per os) or combined with tamoxifen (pellet as above). Mice were sacrificed 1 h after the last treatment and tumors were collected and processed for Western blot.

### RNAseq data generation and analysis

Total RNA was extracted from cell pellets using Trizol Reagent (Thermo Fisher) according to the manufacturer’s instructions. RNA-seq libraries were generated using TruSeq RNA Sample Prep Kit v2 (Illumina) according to manufacturer’s recommendations. The high-throughput sequencing was carried out on a NextSeq 500 (Illumina) using 75 nucleotides single end mode. Xenome software^18^ was used to remove mouse reads. Human associated reads were analyzed on a SeqBox^19^. Demultiplexing (bcl2fastq Illumina tool version 2.17.1.14-2), counts generation using STAR (version 2.5) /RSEM (version 1.3.0) and differential gene expression analysis with DESeq2 (version 1.14.1, adjusted P-value < 0.1 and |log2 fold change|≥ 1) were all performed within the SeqBox framework. Hierarchical clustering was done using Morpheus at Broad (https://software.broadinstitute.org/morpheus/). Ingenuity Pathway Analysis (IPA) (Qiagen) was used for functional characterization of differentially expressed genes. Using the edgeR package we carried out a differential expression analysis between TCGA patients with high levels of gene expression of BCL2 and patients with low levels of gene expression of BCL2 (third and first tertile); we then compared the differentially expressed genes with those differentially expressed in A1 late passage subline.

### Statistical analysis

Student’s *t* test was used to compare tumor volumes, mammosphere productions and RT-PCR expression levels. For tumor growth curves, significantly different tumor volumes at each single time from week 5 onwards are collectively indicated by symbol on the curve. Mantel–Haenszel test was used to compare survival time to 1 cm^3^ tumor volume. Linear regression analysis was used to assess the correlation between tumor doubling time and in vivo passages, and the correlation between the number of in vivo passages and the expression of BCL2. To study the responsiveness to targeted therapies, groups of mice receiving implants from the same PDX were randomized as follows: implants reaching the threshold volume of 10 mm^3^ were alternatively assigned to treated or to untreated group. According to the principles of 3 Rs^20^, the number of mice per group was kept at the minimum necessary to reach statistical significance, depending on the effectiveness of each treatment tested.

### Ethics approval and consent to participate

Human samples were collected after patients gave their informed consent, as indicated in the protocol approved and authorized by the local Ethics Committee (Bologna CE-BI, study number: 14100/CE 2014; prot. N.:964/CE). All methods were performed in accordance with institutional guidelines and Italian law. All human samples and their metadata including relevant clinical data were de-identified before being shared between laboratories involved in this study. All animal procedures were done in accordance with European directive 2010/63/UE and Italian Law (DL 26/2014); experimental protocols were reviewed and approved by the institutional animal care and use committee (“Comitato per il Benessere Animale”) of the University of Bologna (letter 31/1/14), and by the Italian Ministry of Health with letter 687/2015-PR.

## Results

### Engraftment and establishment of orthotopic breast cancer PDX models

The characteristics of our PDX panel (Table 1), obtained from primary untreated breast cancers, mirrored those of clinical records (see also Supplementary Table S1). Tumor take at the first passage was observed in 9/61 cases (15%) (Table 1). Serial transplantation beyond the third in vivo passage is generally considered as a threshold for stabilized PDX^3,12^, along with in vivo growth from frozen tumor fragments. Such conditions were fulfilled by 6 tumors (10%), that were considered transplantable breast cancer PDX models (Table 1). In our hands 17β-estradiol supplement did not produce any advantage in tumor take and growth rate of steroid hormone receptor-positive or -negative tumors (data not shown). In a few mice (3/61) a lymphocytic tumor of human origin appeared shortly after the first implant without any evidence of breast cancer, in agreement with literature^21^. The highest rate of PDX stabilization was obtained in the most aggressive subtypes such as HER2-positive (40%) and triple-negative (17%), followed by luminal B (15%) subtype (Table 1). However, tumor growth parameters (latency and growth rate) of transplantable PDX at the first in vivo engraftment of the surgical sample (data not shown) as well as at the third in vivo passage (Table 1) were independent of the subtype. In contrast, tumor aggressiveness in patient was correlated with PDX take: about half of our clinical cases were grade III invasive carcinomas of no special type (NST), but all established PDX derived from grade III tumors (p < 0.05, Fisher’s exact test).

**Table 1:** PDX from untreated primary human breast cancers of different subtypes.

Subtype	Take	Established^a^	Patient’s code	T	Grading	N	Therapy^b^	PDX latency time^c^	PDX doubling time^d^
Luminal A	1/28 (4%)	0/28 (0%)							
Luminal B	2/13 (15%)	2/13 (15%)	BRS7	T2	III	N0	None	5.0 ± 1.6	10.9 ± 2.1
			BRS18	T1c	III	N1	None	12.0 ± 3.9	17.7 ± 7.2
HER2-Triple positive	2/9 (22%)	1/9 (11%)	BRB15	T2	III	N3A^e^	None	11.3 ± 3.8	24.8 ± 8.1
HER-2 positive	3/5 (60%)	2/5 (40%)	BRB4	T1c	III	N0	None	1.3 ± 0.2	15.3 ± 2.8
			BRS45	T2	III	N2^f^	None	11.3 ± 3.0	21.1 ± 6.8
Triple negative	1/6 (17%)	1/6 (17%)	BRS51	T1c	III	N1^g^	None	4.0 ± 0.0	6.2 ± 0.3

^a^At least 3 passages.

^b^Therapeutic treatment received by patients before surgical removal of the primary tumor from which the PDX was established.

^c^Mean weeks ± SEM of the 3rd in vivo passage (3–7 independent determinations).

^d^Mean days ± SEM of the 3rd in vivo passage (3–7 independent determinations).

^e^11/29 positive lymph nodes.

^f^9/25 positive lymph nodes.

^g^3/30 positive lymph nodes.

### Stability of phenotypic features (clinically relevant molecular markers)

**Figure 1:**
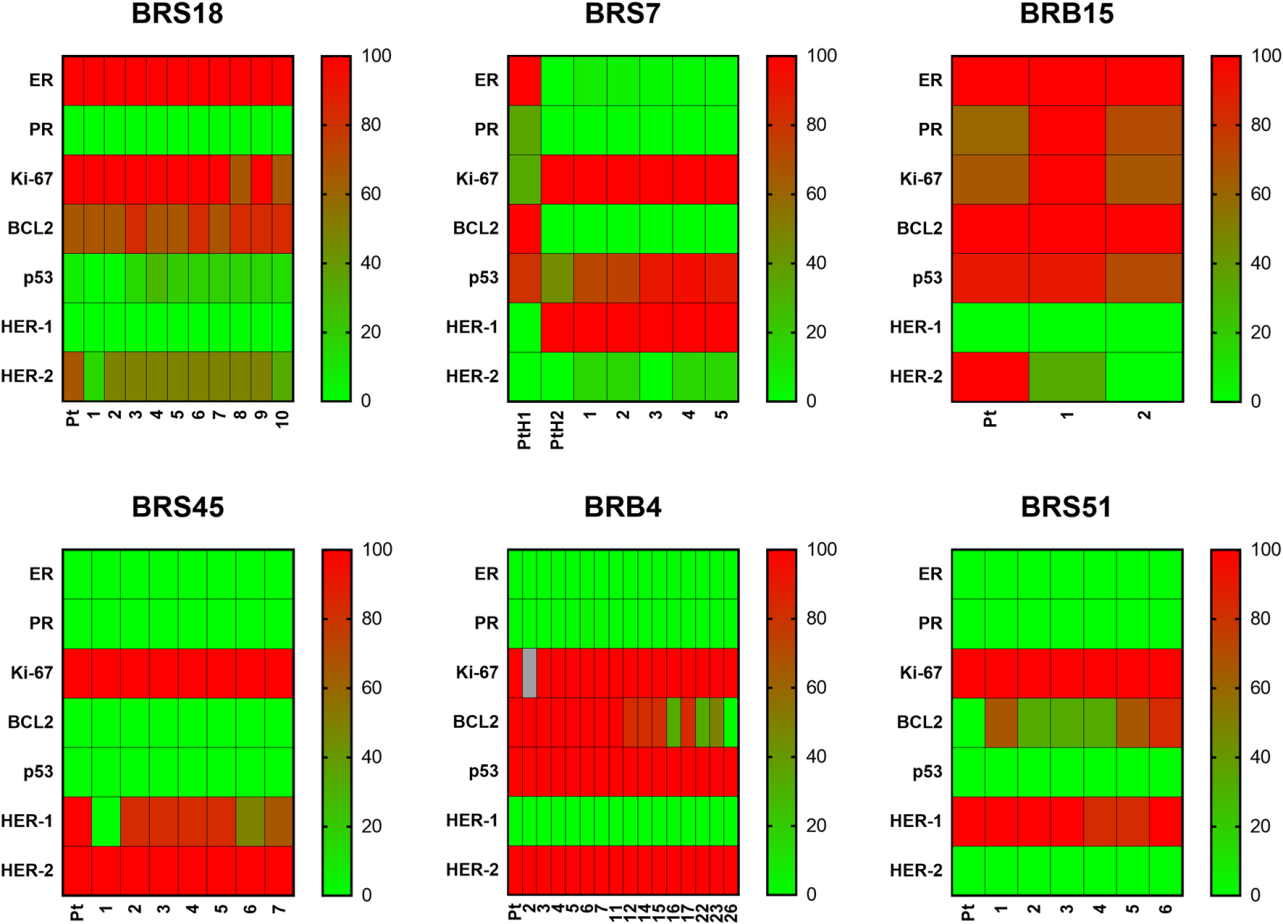
Fidelity and stability of biomarker expression along PDX in vivo passages. Expression data (see Supplementary Table S1 for quantitative data) are represented in a color-code 0–100 scale, arbitrarily attributed to each biomarker (see Supplementary Materials and Methods S1). *Pt* patient’s tumor sample. *Grey* not done. Number of in vivo passages is reported below each graph. For PDX-BRS7 two different histological types were observed in patient’s tumor sample (referred to as PtH1 and PtH2).

The phenotype of original tumors and of corresponding in vivo passages of all PDX models is reported in Fig. 1 and Supplementary Table S2. Most tumors originating PDX showed high proliferative activity. Some heterogeneity among tumors was found for BCL2, p53, HER1 and HER2 expression. Breast cancer PDX models mainly retained biomarkers expression of the subtype of origin, even when transplanted in vivo for ≥ 5 passages. PDX deriving from tumors of highly aggressive subtypes such as HER2-positive (BRB4 and BRS45) or triple-negative (BRS51) appeared to stably maintain the original phenotype in the murine host over passages. The two HER2-positive PDX had different molecular features: PDX-BRS45 expressed HER1 but was negative for BCL2 and p53 expression, while PDX-BRB4 was negative for HER1, positive for BCL2 and had a very high expression of p53. PDX deriving from tumors belonging to less aggressive subtypes but showing a histologically heterogeneous phenotype appeared to go through a selection for the more aggressive variants. BRS7 tumor showed intratumor heterogeneity in the patient both for histology and for most biomarkers expression and was classified as luminal B with a mixed phenotype. BRS7 growth in mice led to the selection of a PDX with low/negative expression of steroid hormone receptors, HER1 positivity, high Ki-67, and BCL2 negativity, more similar to the triple-negative subtype. BRS18 tumor was classified as luminal B but expressed HER2 with score 2 + without gene amplification: in PDX passages it retained expression of ER and HER2. Expression of HER2 full-length (HER2-fl) and of its aggressiveness-related isoform Delta 16 (HER2-D16)^22–24^, were studied by in situ hybridization in two HER2-positive PDX. HER2-fl expression showed score 4 (in a 0–4 score scale, see Supplementary Methods S1) in both PDX, while HER2-D16 isoform expression showed score 2 for PDX-BRB4 (Supplementary Fig. S1) and score 1 for PDX-BRS45 (data not shown). Serial in vivo passaging of the three HER2-expressing PDX did not cause loss of expression of HER2-fl and HER2-D16 nor affected ratio between isoforms (Supplementary Fig. S2). PDX-BRB4 at > 9 in vivo passages showed a slight decrease in HER2 isoform expression, which however remained as high as the control HER2-positive breast cancer cell line BT474.

### Sensitivity to HER2-targeted therapies

**Figure 2:**
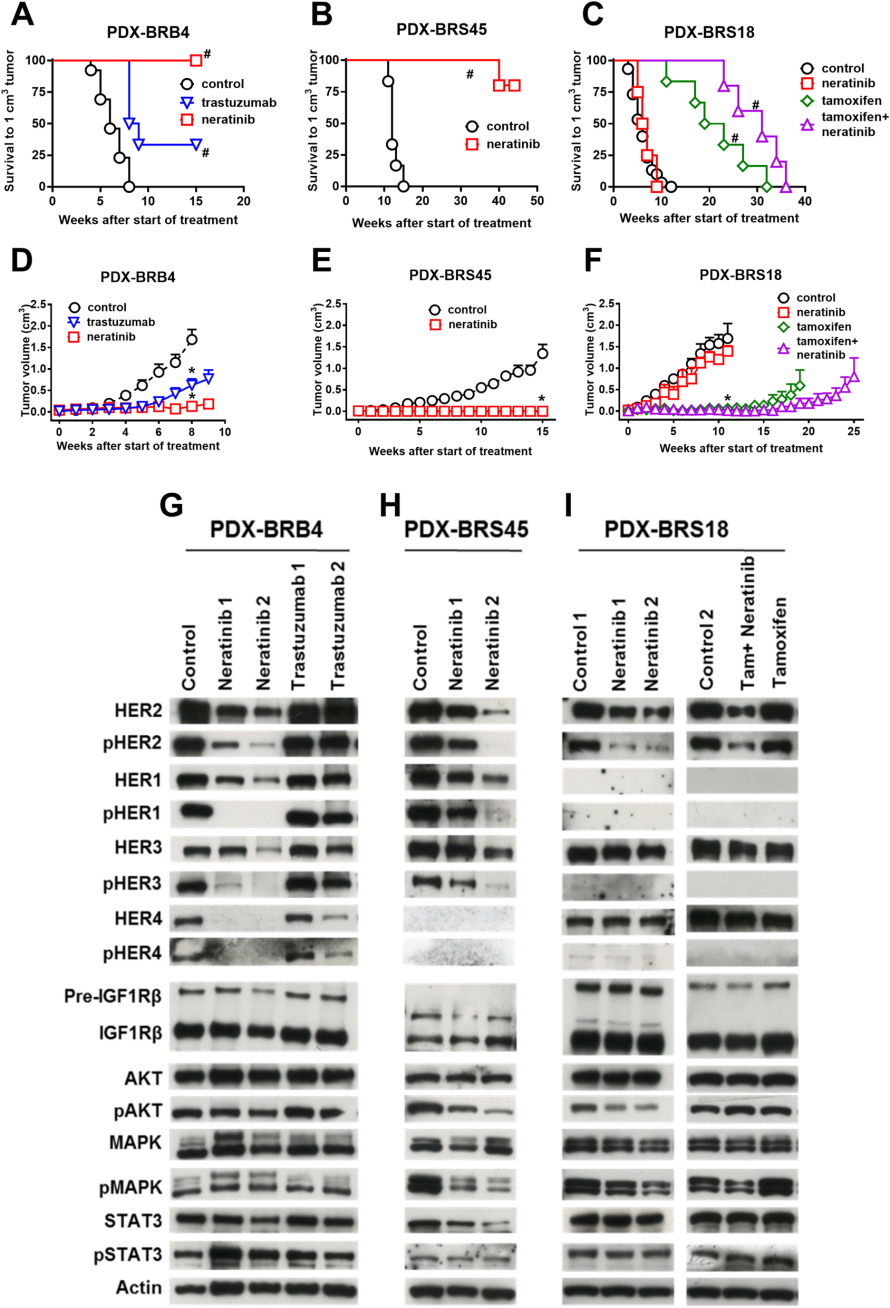
Effects on tumor growth (**A**–**F**) and downstream signaling (**G**–**I**) of HER2 targeted therapies in HER2-expressing PDX. Survival to 1 cm^3^ tumor (Kaplan–Meier analysis, panels **A**–**C**) and tumor growth curves (mean tumor volume and SEM, panels **D**–**F**). Number of in vivo passages and mice: PDX-BRB4, passage 4–6, n = 6–13; PDX-BRS45, passage 5–6, n = 5; PDX-BRS18, passage 7–10, n = 5–20). Significance versus control: *p < 0.05; ^#^p < 0.01. For tumor volumes, significance at each single time from week 5 onwards is collectively indicated by symbol on the curve. Effect of indicated treatments on signaling of PDX-BRB4, PDX-BRS45 and PDX-BRS18 (panel **G**–**I**, in vivo passages 14, 5 and 7–11, respectively). Replicates are from independent mice. Full-length blots are presented in Supplementary Figure S8A.

Our panel of breast cancer PDX with different HER2 expression score and amplification pattern was used to study the effects of two drugs targeting HER2 with different mechanisms, the humanized monoclonal antibody trastuzumab and the small irreversible pan-HER tyrosine kinase inhibitor neratinib. The effect of HER2-targeted therapies against local tumor growth was examined on two HER2 score 3 + PDX (BRB4 and BRS45). Trastuzumab significantly delayed BRB4 tumor growth, while neratinib completely inhibited it (Fig. 2A,D). Neratinib was significantly more effective than trastuzumab (p < 0.05, both in A and in D panel). The complete inhibition of tumor growth by neratinib was confirmed in the PDX-BRS45 (Fig. 2B,E). Trastuzumab caused minor effects on signaling pathways, while neratinib strongly downmodulated all HER family members, both total and phosphorylated isoforms (Fig. 2G,H), as expected^16,25^. Since benefit of HER2 targeted therapies in non-amplified tumors with intermediate levels of HER2 expression (HER2-low), not eligible for HER2-targeted therapies, is still a debated issue^26–28^, we took advantage of our luminal B breast cancer PDX-BRS18 expressing ER and HER2 (score 2 +, without gene amplification), as a model for the evaluation of the impact of HER2-targeted combined therapies. A preliminary in vitro study of primary cell cultures derived from PDX-BRS18 showed that neratinib induced a significant down-modulation of total and phosphorylated HER2 (Supplementary Fig. S3), while it did not modify total nor phosphorylated isoform of AKT and MAPK. When tested in vivo, however, neratinib did not affect tumor growth of PDX-BRS18 (Fig. 2C,F), even though drug treatment decreased both total and phosphorylated HER2 (Fig. 2I). BRS18 did not show HER1 and expressed HER3 and HER4 but not their phosphorylated isoforms. Neratinib did not modify the expression of HER3 and HER4. Strong inhibition of tumor growth was obtained with the ER-targeted tamoxifen treatment. Neratinib combined with tamoxifen treatment gave a slightly increased control of tumor growth compared to tamoxifen alone (Fig. 2C,F). On the whole, in our PDX series neratinib efficacy on controlling tumor growth seems to require the simultaneous downmodulation of all HER family members, while downmodulation of pHER2 alone was not sufficient for growth control of tumors with other driver genes, such as ER.

### Progression studies

**Figure 3:**
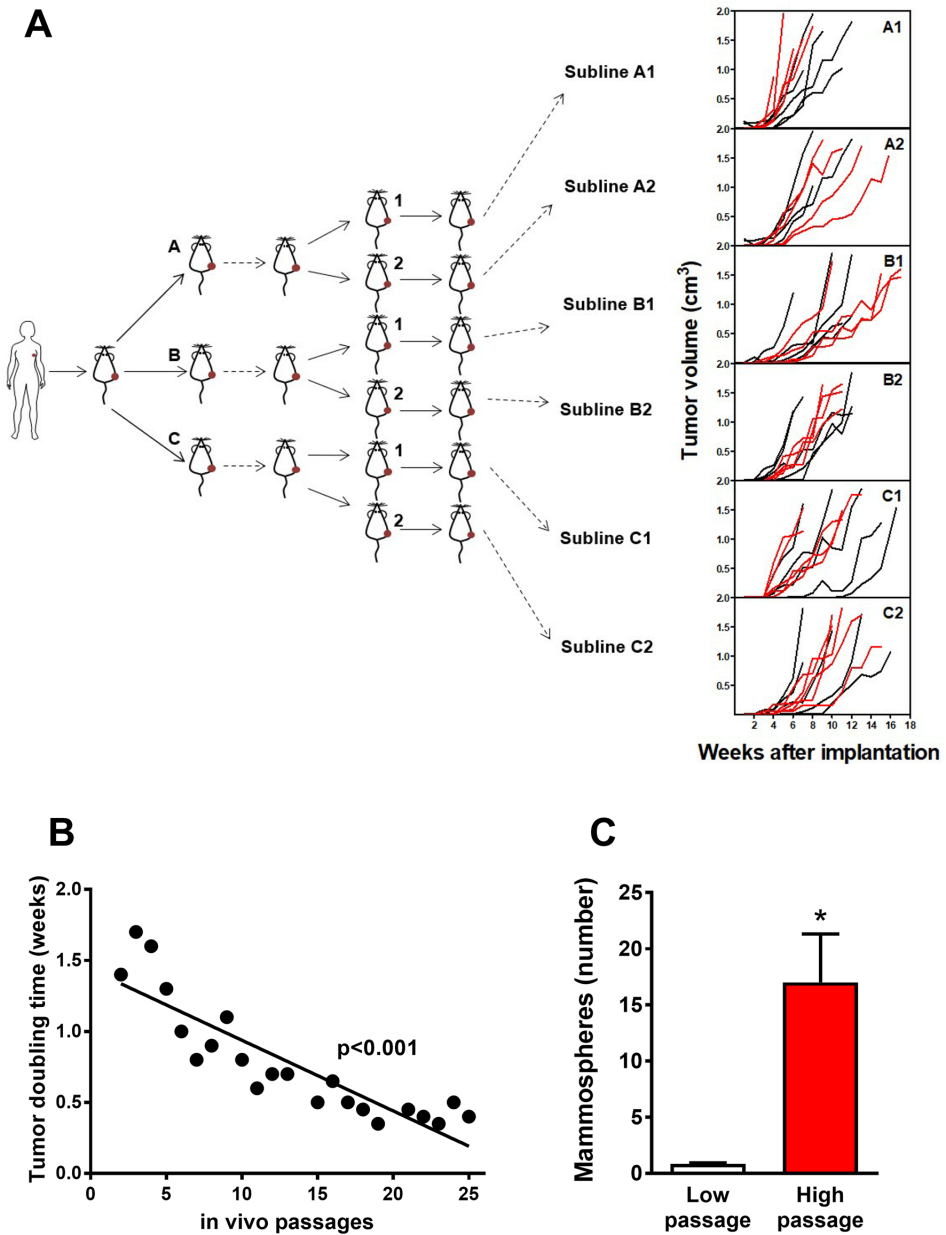
Random progression in sublines of PDX-BRB4. (**A**) Origin of independent sublines of PDX-BRB4 (see “Methods”) and growth kinetics during in vivo passages. Right panels show individual tumor growth curves of sublines at low-passage (passage 4–8, black lines) and high-passage (14–18, red lines). (**B**) Tumor doubling time of subline PDX-BRB4-A1 during long-term in vivo passages (up to approximately 4 years), calculated in the exponential growth phase. Significance of linear regression is shown in the panel. (**C**) Mammospheres formed in vitro by PDX-BRB4-A1 at low (8) and high (23–26) passage. Mean and SEM of 3–4 independent determinations (6 replicates each) are shown. *p < 0.05.

**Figure 4:**
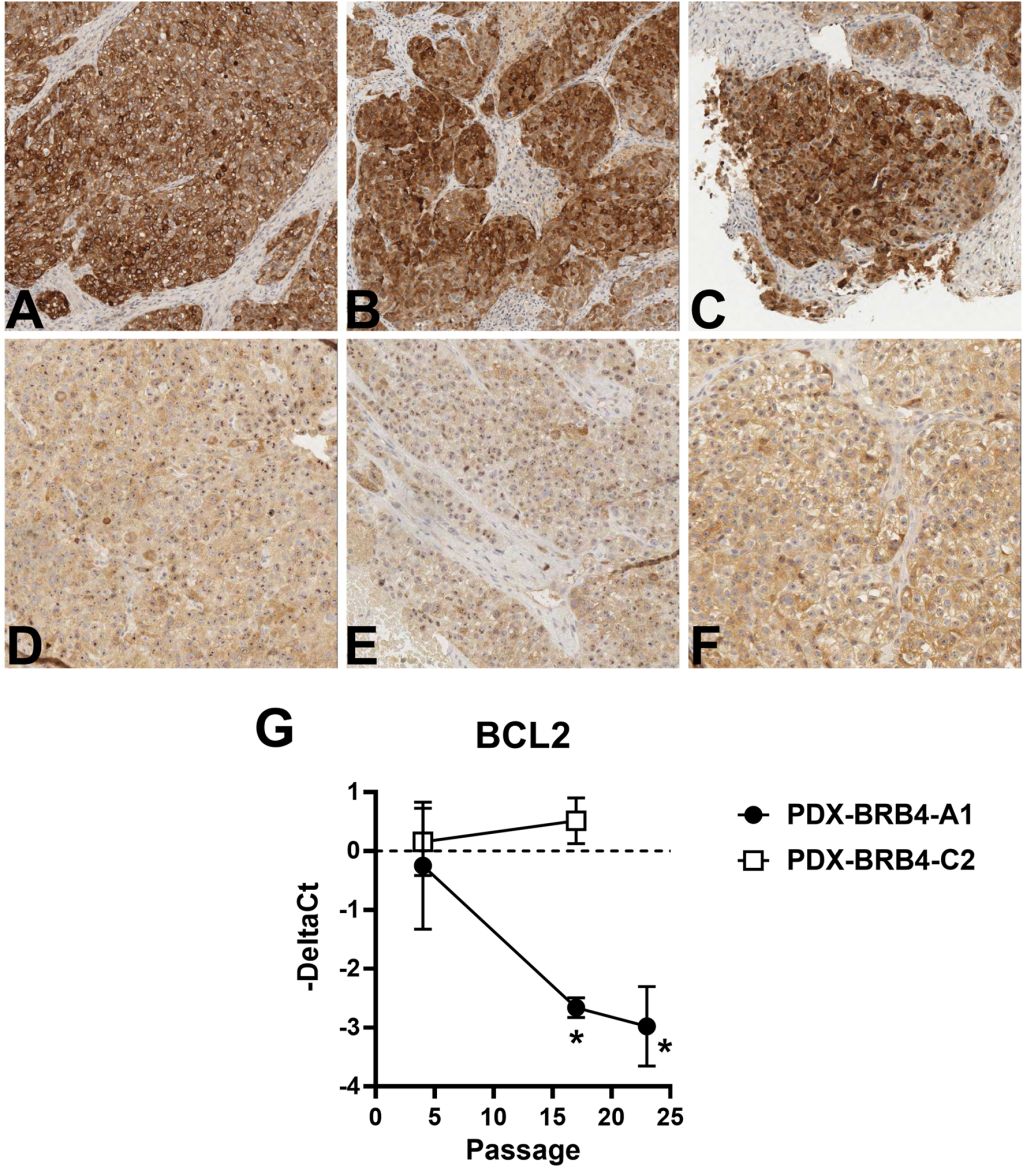
Decrease of BCL2 expression during tumor progression. (**A**–**F**) Immuno-histochemical expression of BCL2 in PDX-BRB4-A1 at low-passage (5–7 passages, panels **A**–**C**) and in its progressed high-passage subline (23–26 passages, panels **D**–**F**). Sections were immunostained with antibodies against BCL2 biomarker. (**G**) RT-PCR expression level of BCL2α in PDX-BRB4 sublines at different in vivo passages. Mean and SEM from three independent replicates is shown. Significance: linear regression of BCL2 in PDX-BRB4-A1 subline, *p* < 0.01; *p < 0.05 high-passage A1 versus low-passage A1 and versus C2.

To investigate PDX tumor progression, HER2-amplified PDX-BRB4 was split after the second passage in six different sublines, which were then re-transplanted separately to analyze random and selective events in long-term evolution (Fig. 3A). One out of six sublines (named PDX-BRB4-A1), passaged up to 25 times along approximately 4 years, progressively acquired a significantly increased tumor growth rate though maintaining biomarker profile (Fig. 3B and Supplementary Table S2). High-passage A1 subline showed an increased ability to form mammospheres in vitro (Fig. 3C), which showed a CD24^low^/CD44^high^ phenotype (data not shown), denoting an enriched cancer stem cell phenotype. High-passage PDX-BRB4-A1 subline showed a progressively decreased BCL2 expression by immunohistochemistry, with a shift from highly positive to intermediate/negative, confirmed by RT-PCR analysis (Fig. 4). Macroscopic and molecular evidence of metastatic spread was absent in low-passage PDX-BRB4-A1 subline, in contrast metastatic cells were sporadically detected in the lungs from high-passage PDX-bearing mice. To better evidence the different metastatic ability, cells dissociated from in vivo passaged PDX-BRB4 sublines were injected i.v. to evaluate hematogenous metastasization. Low-passage A1 did not produce metastatic deposits in the lungs, whereas high-passage progressed A1 subline gave rise to overt lung metastases (Table 2). Low- and high-passage A1 sublines of PDX-BRB4 differed in the ability to grow in cultures: low-passage PDX cultures stopped growing and underwent senescence more rapidly than high-passage A1 (Table 2 and Supplementary Fig. S4). No difference was found between low and high passages of non-progressed sublines of PDX-BRB4, which rapidly underwent senescence.

**Table 2:** Functional progression of PDX-BRB4-A1 subline.

PDX-BRB4 subline	In vivo passages (range)	Tumor doubling time (weeks)	Lung metastases^e^ (mice with metastases/total number of mice)	Time to in vitro senescence (days, mean and SEM)
A1	3–5	1.5 ± 0.1	0/4	52.0 ± 4.4
A1	15–24	0.5 ± 0.03^a,b^	4/4	145.8 ± 16.9^a,b^
C2	3–5	1.4 ± 0.6	nd^c^	50.3 ± 22.8
C2	14–17	0.8 ± 0.1	0/3^d^	11.7 ± 5.0

^a^*P* < 0.05 at least vs A1 3–5 passages (Student’s *t* test).

^b^*P* < 0.05 at least vs C2 14–17 passages (Student’s *t* test).

^c^Not done.

^d^Negativity confirmed by molecular assay.

^e^After i.v. injection of dissociated cells.

### Development of resistance to HER2-targeted agents

**Figure 5:**
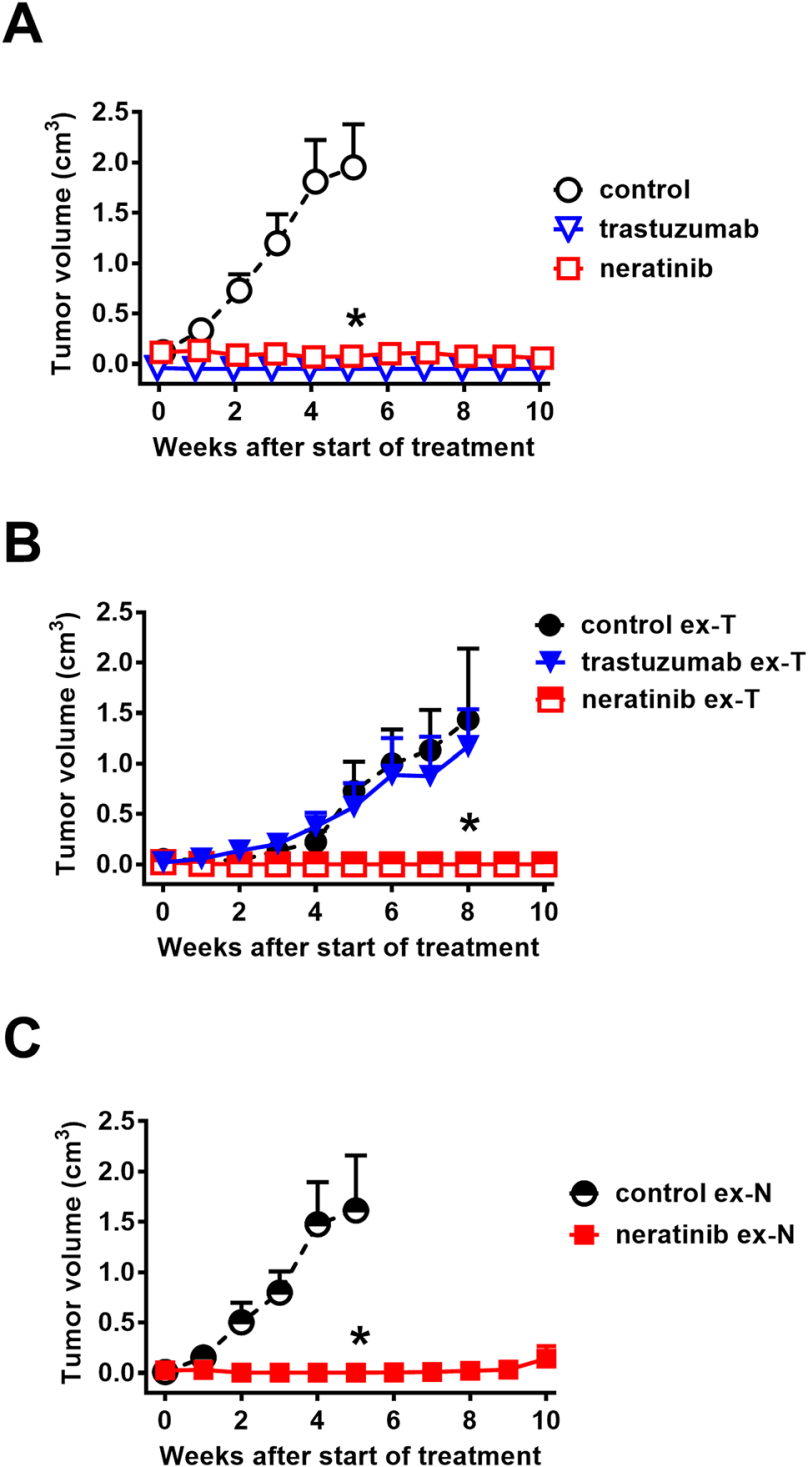
Resistance/sensitivity to different HER2-targeted treatments by PDX-BRB4 variants. (**A**) Sensitivity to HER2-targeted treatments of progressed PDX-BRB4-A1 subline (number of passages and mice per group: 21–27, n = 3–10). (**B**) PDX-BRB4 implants from xenografts chronically pre-treated with trastuzumab (ex-T) are still responsive to neratinib (number of passages and mice per group: 6–10, n = 4). (**C**) PDX-BRB4-A1 implants deriving from xenografts chronically pre-treated with neratinib (ex-N) are still responsive to neratinib (number of passages and mice per group: 22–23, n = 4). Significance versus control: *p < 0.05.

The development of drug resistance is a further hallmark of advanced tumor progression affecting both conventional and targeted therapies. Treatment of high-passage progressed PDX-BRB4-A1 tumors with trastuzumab or neratinib showed a strong sensitivity to both targeted agents (Fig. 5A), even more pronounced than that of low passages (compare Fig. 5A with 2D), thus indicating that in this system in vivo progression did not entail the emergence of drug-resistant populations in the absence of drug treatments. To analyze the development of resistance after long-lasting in vivo drug treatment, PDX-BRB4-A1 was serially implanted in mice treated with either trastuzumab or neratinib (> 8 months). Tumors previously exposed to trastuzumab (ex-T) were no longer inhibited by trastuzumab itself, even though HER-2 expression was not significantly modified (Supplementary Fig. S7), but were completely inhibited by neratinib (Fig. 5B); on the contrary, tumors previously exposed to neratinib (ex-N) remained sensitive to neratinib (Fig. 5C), on the whole suggesting that neratinib could be indicated for the treatment of cases that develop trastuzumab resistance after the treatment.

### RNAseq analysis of progressed A1 subline of PDX-BRB4

**Figure 6:**
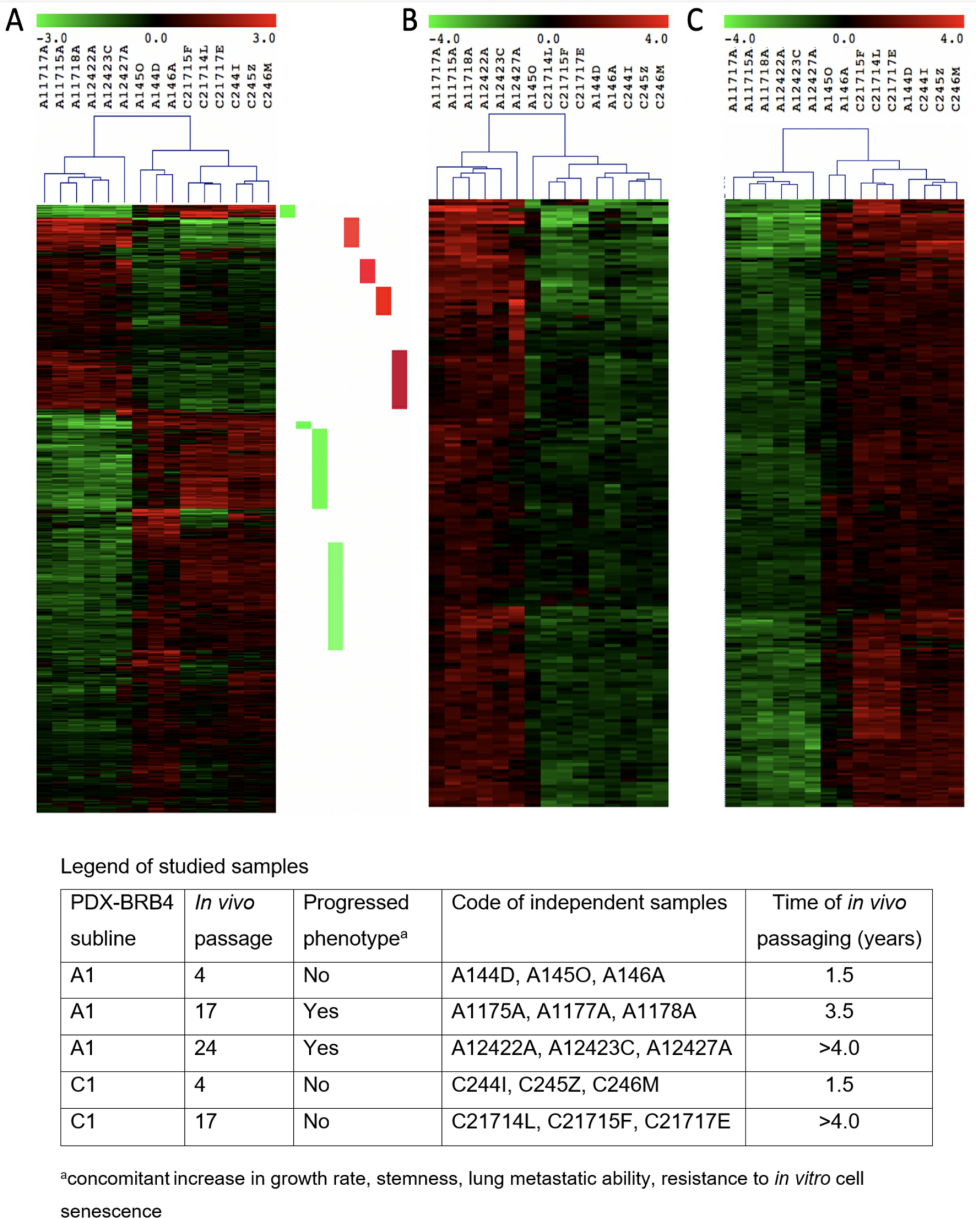
Hierarchical clustering (Euclidean distance, average linkage) of differentially expressed genes in progressed subline (see Legend Table enclosed for codes of independent samples examined along with their progression phenotypes). (**A**) 834 DE genes detected comparing progressed (passage 17) with non-progressed (passage 4) A1 subline of PDX-BRB4. Red and green lateral bars indicate the gene subsets showing homogeneous progressed phenotype (passages 17 and 24) with respect to non-progressed low-passage 4 (either subline) or 17 (C1 subline). (**B**) 193 DE genes up-modulated in progressed A1 subline (passage 17 and 24, see red bars of panel **A**). (**C**) 288 DE genes down-modulated in progressed A1 subline (passage 17 and 24, see green bars of panel **A**).

**Figure 7:**
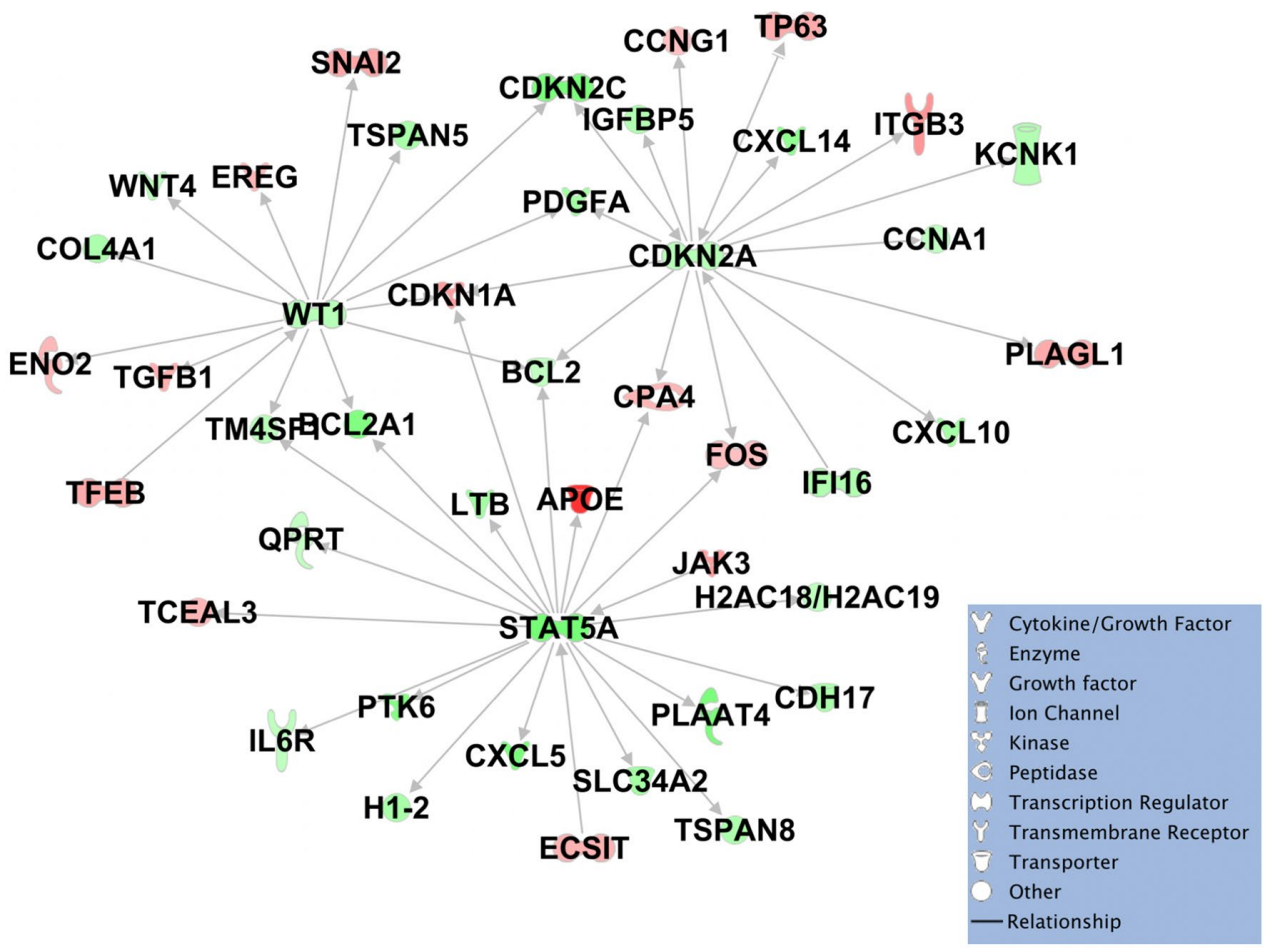
IPA sub-network showing that BCL2, which is down-modulated in progressed A1 subline (passage 17 and 24), is connected to three main hub genes also down-regulated (CDKN2A, STAT5A and WT1).

The acquirement of a progressed phenotype by the PDX-BRB4-A1 subline from low to high passages (as defined based on the concomitant increase in growth rate, stemness, lung metastatic ability and resistance to in vitro cell senescence) was studied through the analysis of transcription profiles, in comparison to low and high passages of a non-progressing subline (C1). Differential expression analysis (adjusted P-value < 0.01 and |log2 fold change|≥ 1) was performed comparing progressed A1 subline at passage 17 with respect to those at passage 4. RNAseq analysis was done on triplicate independent samples per group (see Legend of samples, Fig. 6). A total of 834 differentially expressed genes (DE) were detected (Supplementary Table S3A). The transcription profiles of A1 subline at passage 24 were nearly superimposable to those of A1 at passage 17 (Supplementary Fig. S5) and were not used in the differential expression to keep comparison between the two groups balanced. Hierarchical clustering (Euclidean distance, average linkage) (Fig. 6A) of the 834 DEs was used to identify the subset of genes characterizing the progressed phenotype, by comparing progressed phenotype (passages 17 and 24 of A1 subline) with respect to non-progressed samples (passage 4 of A1 subline and passages 4 and 17 of C1 subline). Only genes showing an expression difference between progressed phenotype and all other samples were kept: 193 DE genes up-modulated (UP) in progressed phenotype (Fig. 6B) and 288 DE genes down-modulated (DW) in progressed phenotype (Fig. 6C). The 481 DE genes (193 UP and 288 DW) detected by hierarchical clustering (Supplementary Table S3B) were loaded in an IPA pathway. The genes were then connected using only direct interaction between genes, e.g. an article describes that gene *X* is affecting gene *Y* activity (Supplementary Fig. S6). Within this network we identify that BCL2, which is down-modulated in progressed phenotype, is connected to three main hub genes also down-regulated: CDKN2A, STAT5A and WT1 (Fig. 7). Looking to genes up- and down-modulated in the epithelial-to-mesenchymal transition (EMT)^29,30^, we found that progressed A1 subline compared to non-progressed cell variants (Supplementary Table S3B) had increased expression of some genes usually up-modulated in EMT (such as COL6A3, ITGB3, SNAI2/SLUG, TGFbeta1 and BMP2) and decreased expression of some genes usually down-modulated in EMT (such as CLDN10, CLDN3 and BMP5). However, the main genes driving or inducing EMT (such as Twist, ZEB1, ZEB2, SNAIL, E-CAD, Vimentin, COX2 and NOTCH) were not differentially expressed (and therefore were not included in DE genes listed in Supplementary Table S3). Such expression pattern suggests that progressed A1 subline is undergoing a partial EMT, as also shown by the maintained epithelial cell morphology.

We compared the transcriptional profiles of TCGA Her2 + patients with BCL2 down modulation with the transcriptional profile of late passage A1 subline with BCL2 downmodulation (Supplementary Table S3B). About 10% of differentially expressed genes in the PDX were concordantly differentially expressed in clinical samples (genes are marked with a * in Supplementary Table S3B).

## Discussion

In our study, the common features of breast cancers of different subtypes that gave rise to PDXs were high grade, high proliferation, and absent/limited hormone dependence. The biomarkers typical of each subtype were maintained for five to ten in vivo passages. The highest rate of PDX stabilization was found for HER2-positive tumors (40%). We obtained two score 3 + HER2-positive PDX showing differences in relevant molecular aspects, such as p53 and BCL2 expression, and a score 2 + HER2-not amplified PDX.

Even if most breast cancer cell lines in the literature were established from pleural effusions, ascites and other metastatic sites, and most breast cancer PDX were derived from primary tumors, it is becoming evident that the PDX generation procedure selects for the most aggressive tumor variants^14^. Significant differences in patient cohorts and tumor features might account for variability among studies in tumor take and stabilization. The major determinant of PDX tumor take is the aggressive phenotype of the tumor^14^, whereas the use of severely immunodeficient mice, the implantation in orthotopic sites, or the hormonal conditioning of the host play minor roles^31^. New approaches are needed to increase breast cancer PDX take. Patient cohorts with the higher frequency of aggressive subtypes have shown higher rates of tumor take and stabilization and in most studies PDX mainly derived from aggressive subtypes, such as triple-negative, luminal B or HER2-positive subtypes.

In two HER2-positive breast cancer PDX carrying amplified HER2 genes and displaying HER2 as driver mutation, neratinib as single treatment significantly inhibited tumor growth and increased survival. Despite the generation in vitro of the neratinib-resistant variant of HER2-positive cell lines reported in the literature^32^, in our PDX models no appearance of resistance to neratinib in vivo was seen and tumor relapsing after drug interruption when implanted in other mice displayed again neratinib sensitivity.

Sensitivity to standard-of-care treatments has been studied and compared with the response in patients showing high concordance of data and even new targeted therapies and their mechanisms of action have been evaluated in different subtypes of breast cancer PDX models. Despite several efforts, metastatic growth and tumor progression per se or after therapeutic treatments have been rarely obtained and even the selection for metastatic PDX variants has been not at all satisfactory^33^. For both HER2-positive PDX we provided evidence of sensitivity to HER2-targeted therapies with the greatest tumor growth inhibition obtained with the pan-HER kinase inhibitor neratinib. For PDX-BRB4 we developed a trastuzumab-resistant variant that retained strong sensitivity to neratinib tumor growth inhibition. The acquired trastuzumab resistance did not appear to depend on the loss of HER-2 expression, as the levels of p185 detected by flow cytometry were only slightly decreased (Supplementary Fig. S7); we are currently investigating various possible mechanisms of trastuzumab resistance, including mitogenic pathways mediated by other members of the HER/ErbB family sensitive to neratinib, or the involvement of Pleckstrin Homology Like Domain Family A Member 1 (PHLDA1), which is expressed by PDX-BRB4 (data not shown) and is reported to mediate drug resistance in tyrosine kinase-driven cancers^34^.

In our study the long-term in vivo re-transplantation for up to 25 passages of a HER2-amplified PDX allowed the random emergence of a progressed phenotype. Among 6 different sublines, only one (PDX-BRB4-A1) showed the shift toward a progressed phenotype, with faster in vivo growth, acquirement of metastatic ability after i.v. injection, enriched stem cell phenotype and lower in vitro cell senescence. Such a shift was defined as functional progression since it was not linked to alterations in molecular parameters of the breast cancer subtype. The sensitivity to HER2-targeted therapy was not lost by progressed PDX-BRB4-A1 subline but even increased.

Tumor progression is based on the genomic instability of tumor cells, which leads to the generation of variants subject to environmental selection. In the case of PDX-BRB4, the progression towards malignancy of just one subline suggests that malignant variants were not pre-existent, or were very rare in the original population, otherwise we would have obtained tumor progression in a higher proportion of sublines. The events driving genomic instability are usually ascribed to caretaker tumor suppressor genes, such as p53 and BRCA1; in the case of p53 the original BRB4 tumor was 100% positive by immunohistochemistry (Supplementary Table S2), which is suggestive of mutations yielding dominant-negative p53 subunits; we will investigate the possible involvement of other caretaker genes, such as BRCA1 and BRCA2.

HER2-amplified PDX-BRB4 showed a progressive decrease of BCL2 expression after long-term in vivo passage, being almost negative after ≥ 22 passages. A similar phenomenon also occurred with the luminal B/mixed phenotype PDX-BRS7, where the BCL2-negative variant rapidly emerged.

High level expression of BCL2 in breast cancer is associated with a better prognosis^35–38^. Therefore, the downregulation observed during in vivo passages of our PDX could be linked to the selection of more aggressive variants, showing a positive prognostic role for the biomarker BCL2, as observed in clinical studies.

BCL2 gene product (mainly its alpha isoform) plays an anti-apoptotic role^39^. The paradox of low BCL2 levels associated with a bad prognosis in breast cancer could be related to Beclin-1 inhibition. Beclin-1 induces autophagy leading to survival promotion and maintenance of cancer stem cells^39^. BCL2 is a negative regulator of Beclin-1 and can inhibit its pro-tumorigenic effects inducing cell senescence and growth arrest^39^. Our data highlighted a complex interaction of BCL2 with CDKN2A, STAT5 and WT1, concordantly downregulated in the progressed A1 subline. In our progressed model low levels of BCL2 were not related to therapy resistance, as also reported in clinical studies^40^. On the other hand, a high expression of WT1 in breast cancer is clinically associated to increased malignancy, bad prognosis, lower responsivity to therapies and to a mesenchymal phenotype. Breast cancer cell lines with mesenchymal *versus* epithelial phenotypes were reported to show divergent effects when subjected to silencing or hyperexpression of WT1^41^, suggesting that the epithelial-to-mesenchymal transition (EMT) could play a role in the way towards bad prognosis and lower responsivity to therapies. In our study, the progressed PDX-BRB4-A1 subline underwent an only partial EMT. A1 progressed subline showed increased malignancy and stemness but maintained epithelial morphology and did not acquire resistance to therapy with trastuzumab or neratinib. A higher stemness and metastatic ability could be linked to early events of EMT, while resistance to therapies could require further steps of the transition.

Obtaining models of human tumor progression is an open challenge for researchers and PDX models of progression with the acquirement of increased metastatic ability are very rare. Our data suggest that progression can develop randomly along several months or years, like in humans. Clonal dynamics, evaluated at single cell resolution, in xenograft lines by serially transplantation of organoid suspension from breast cancer, seems to be based mainly on deterministic mechanisms^42^. These findings suggest that, due to intratumoral heterogeneity, studies on progression and onset of drug-resistant variants could take advantage of the propagation of several sublines from each single patient.

## Conclusion

Our data enlighten the pros and cons of breast cancer PDX models. Their use as an individual patient’s avatar to set up in vivo personalized treatments is frustrated by the very low take rate, and by the onset of lymphoma in a subset of them. Concerning the stability of PDX over time, it could be a double-edged sword. On the one hand, stability for five to ten in vivo passages of functional (such as growth rate, metastatic ability, senescence, stemness) and clinically relevant biomarkers (see for example HER2, EGFR/HER1, hormone receptors, p53, Ki-67) allows the study of mechanisms and determinants of responsiveness or resistance to drugs. On the other hand, stability cannot allow to predict or make faster the tumor progression that will occur in the patient. A random progression occurred in a breast cancer PDX, even in the absence of treatment, but only after sequential in vivo passages for years, a time span like those observed in the progression of human tumors in patients. Such progression, however, was not linked to alterations in molecular parameters of the breast cancer subtype. The loss of BCL2 and the concomitant downregulation of its three main hub genes occurring in the progressed subline, with increased stemness and partial EMT, could allow a better understanding of prognostic factors and designing of new therapeutic approaches. Up to now the take rate of breast cancer PDXs is insufficient to allow the design of precision therapies tailored on each patient, unless PDX derivation from breast cancer becomes more efficient. Finally, the use of PDX for studies on the progression of individual patients will require a great effort to expand sublines and years to allow the appearance of progressed sublines in absence of therapeutic or microenvironmental manipulations.

## Supplementary Information

Supplementary Information 1.

Supplementary Information 2.

## Data Availability

All data generated or analyzed during this study are included in this published article and can be shared by contacting the corresponding authors.
