# Supplementary material for: Early stability and late random tumor progression of a HER2-positive primary breast cancer patient-derived xenograft
Source: Sci Rep. 2021 Jan 15;11:1563. doi: 10.1038/s41598-021-81085-y (PMC7810859; doi:10.1038/s41598-021-81085-y)

Supplementary info

Early stability and late random tumor progression of a HER2-positive primary breast cancer patient-derived xenograft

Lorena Landuzzi[1#], Arianna Palladini[2#], Claudio Ceccarelli[3], Sofia Asioli[4], Giordano Nicoletti[1], Veronica Giusti[2], Francesca Ruzzi[2], Marianna L. Ianzano[2], Laura Scalambra[2], Roberta Laranga[2], Tania Balboni[2], Maddalena Arigoni[5], Martina Olivero[,6,7], Raffaele A. Calogero[5], Carla De Giovanni[2], Massimiliano Dall'Ora[2], Enrico Di Oto[4], Donatella Santini[8], Maria Pia Foschini[4], Maria Cristina Cucchi[9], Simone Zanotti[10], Mario Taffurelli[10], Patrizia Nanni[2§], and Pier-Luigi Lollini[2§*].

[1]Laboratory of Experimental Oncology, IRCCS Istituto Ortopedico Rizzoli, Bologna, Italy.
[2]Laboratory of Immunology and Biology of Metastasis, Department of Experimental, Diagnostic and Specialty Medicine (DIMES), University of Bologna, Viale Filopanti 22, I-40126 Bologna, Italy. [3]Laboratory of Oncologic Immunocytopathology, DIMES, St. Orsola-Malpighi Hospital, University of Bologna, Via Massarenti 9, 40138, Bologna, Italy.
[4]Department of Biomedical and Neuromotor Sciences, University of Bologna, Unit of Anatomic Pathology "M. Malpighi", Bellaria Hospital, Via Altura, 3, 40139, Bologna, Italy.
[5]Department of Molecular Biotechnology and Health Science, University of Torino, Torino, Italy. [6]Department of Oncology, University of Torino, Italy.
[7]Candiolo Cancer Institute-FPO, IRCCS, Candiolo, 10060 Torino, Italy.
[8]Pathology Unit, St. Orsola-Malpighi Hospital, University of Bologna, via Massarenti 9, 40138, Bologna, Italy. [9]Unit of Breast Surgery, Bellaria Hospital, AUSL Bologna. [10]Department of Medical and Surgical Science, Bologna University- Breast Unit Sant'Orsola Hospital, Bologna.

[#]Lorena Landuzzi and Arianna Palladini contributed equally.

[§]Patrizia Nanni and Pier-Luigi Lollini jointly supervised this work.

*Correspondence and requests for materials should be addressed to Pier-Luigi Lollini (email: pierluigi.lollini@unibo.it).

Supplementary Methods

Histology and immunohistochemistry

Human tumors or xenografts were fixed in 10% neutral buffered formalin solution (Sigma-Aldrich) for 12-24 hours at room temperature (RT), and processed using the formalin/ethanol/isopropanol/paraffin protocol by a hybrid tissue processor (Logos, Milestone Srl, Sorisole IT) to obtain paraffin blocks. Serial sections of formalin-fixed, paraffin-embedded (FFPE) tissue were cut, collected on adhesive glass slides (Tom-11, Matsunami Glass Ind., Ltd Osaka Japan) and air dried for at least 30 min at RT. Immunostaining was performed on FFPE sections in an automated Benchmark Ultra Autostainer (Ventana Medical Systems, Inc., Tucson, Arizona, USA). The immunologic reaction was visualized using the Ventana UltraView DAB Detection kit according to the manufacturer's instructions. Ready-to-use primary antibodies recognized: BCL2 (clone SP66), estrogen receptor (ER, clone SP1), progesterone receptor (PR, clone 1E2), Ki-67 (clone 30-9), HER2 (Pathway clone 4B5), HER1/EGFR (clone EP38Y, rabbit); p53 (clone DO7). All the antibodies were purchased from Ventana, except anti-HER1/EGFR, purchased from Thermo Fisher Scientific, Fremont USA or Abcam, Cambridge, UK. Nuclear immunostaining for ER, PR, Ki-67 and p53 was quantified using a semi-automated image analysis system (Image-Pro plus v.5.0.1, Media Cybernetics Inc., Rockville, USA) on at least 30 randomly selected 200x microscopic fields (2.000 cells) and expressed as percentage of immunostained neoplastic population. ER and PR were considered positive if ≥10% of neoplastic population was immunostained; Ki-67 values were classified as follows: Low ≤ 20%, Intermediate >20≤30%, High > 30% of immunostained cells, according to St Gallen consensus 2015 suggestions[1]. HER2 expression was evaluated following ASCO/CAP 2013 recommendations[2]. HER1/EGFR

and BCL2 expression was semi-quantitatively evaluated by examining all the neoplastic population at 100x and classified as follows: Negative < 10%; Intermediate ≥10%<30%; Positive ≥30% of immunostained neoplastic cells. Appropriate positive and negative controls were included in each run, furthermore all stained sections included non-tumor mouse cells, such as endothelial cell, myopericytes and fibroblasts which were invariably negative. To represent data in a more intuitive way, a color-code 0 to 100 scale was arbitrarily attributed to each biomarker as follows. Ki-67: high/positive (>30%), 100; intermediate (>20-≤30%), 66; low (≤20%), 33; negative, 0. BCL2 and EGFR/HER1: high/positive (≥30%), 100; intermediate (≥10-<30%), 66; low (≤10%), 33; negative, 0. HER2: 3+/amplified, 100; 2+, 66; 1+, 33; negative, 0. For ER, PR and p53 the median percent value was used.

FISH analysis

Identification of the HER2 status was performed using a dual-colour FISH analysis as follows: standard 4- to 5-μm sections of FFPE tissues were incubated at 56°C for 2 hours in a dry oven, deparaffinized by washing in 3 steps in Bioclear (Natural terpenes-based clearing agent; Bio-Optica, Milan) for 15 minutes each, dehydrated in 2 steps of 5 minutes each in Dehyol absolute (alcoholic mixture made up of ethanol; Bio-Optica) and air dried at RT. After incubation in 2× saline sodium citrate buffer (2× SSC; pH 7.0) at 75°C for 12 minutes, the sections were digested with proteinase K (0.25 mg/mL in 2× SSC; pH 7.0) at 45°C for 12 minutes, rinsed in 2× SSC (pH 7.0) at RT for 5 minutes, and dehydrated using ethanol in a series of increasing concentrations (70%, 85%, and 100%) for 2 minutes each. According to the manufacturer's instructions, on HER2/CEP17 dual colour, the probe (KREATECH Diagnostics, Amsterdam, The Netherlands) was applied on the area selected based on the presence of immunohistochemically positive cells.

The hybridization area was covered with a coverslip and sealed with rubber cement. The slides were incubated at 75°C for 10 minutes for co-denaturation of chromosomal and probe DNA and then at 37°C for 20 to 24 hours to allow hybridization. Post-hybridization washes were performed in NP40 0.5%/SSC 2× (pH 7.0- 7.5) at 75°C for 2 minutes and in 2× SSC for 2 minutes at RT. After the samples were dehydrated in ethanol as above, 4′,6′-diamidino-2-phenylindole suspended in antifade diluents 1 µg/mL (KREATECH Diagnostics, Amsterdam, The Netherlands) was applied for chromatin counterstaining. FISH analysis was carried out using an Olympus BX61 epifluorescence microscope (Olympus, Melville, NY).

Cell cultures from PDX

Primary cell cultures were set up from mechanically dissociated tumors in Primaria tissue culture flasks (Falcon, Becton Dickinson, USA) in HuMEC complete medium + 20% FBS, all from Invitrogen (UK). All cells were maintained at 37°C in a 7% CO_2 atmosphere with frequent medium renewal. Cell cultures were splitted using a 0.025% trypsin - 0.001% EDTA solution (Invitrogen, UK). To study mammosphere production, cells were seeded in Ultra Low Attachment Surface (Corning, Costar, NY, USA) Polystyrene 6-well plates, 4000 cells per well in 4 mL Mammocult (StemCell Technologies, Canada) complete medium without FBS. After 6-8 days of culture, mammospheres were counted in 6 replicated wells at 31.25-125× magnification. Mammospheres were collected and dissociated by incubation in Trypsin-EDTA to obtain single cell suspensions for direct immunofluorescence with anti-hu-CD24AF488 (clone ML5, BioLegend Cat# 311108, RRID:AB_528782) and anti-hu-CD44PE (clone IM7, BioLegend Cat# 103007, RRID:AB_312958). Cytofluorometric analysis was performed by FACScan (Becton Dickinson, USA) and CyFlow Space (Sysmex Partec,

Germany) instruments. The cell senescence process was detected using the Senescence Cells Histochemical Staining Kit (Sigma-Aldrich, St. Louis, MO, USA). This assay is based on a histochemical stain for β-galactosidase activity at pH 6, a well-known senescence marker. Cells were fixed and processed according to the protocol reported by the kit.

Gene expression

Total RNA was extracted from snap-frozen tumor samples or lungs and ovaries, using TriZol "Total RNA isolation reagent" (Thermo Fisher Scientific, MA, USA) and gentleMACS Octo Dissociator (Miltenyi, Germany). HER2 *full length* and *Delta*16[3,4] gene-expression was analyzed by Real Time-PCR (RT-PCR) using the following primers HER2 full length, Dir: GTGTGGACCTGGATGACAAGGG, Rev: GCTCCACCAGCTCCGTTTCCTG; HER2 Delta16, Dir: CACCCACTCCCCTCTGAC, Rev: GCTCCACCAGCTCCGTTTCCTG. TATA binding protein (TBP) genes were chosen as housekeeping genes[5] using the following primers: human, Dir: AGAACAACAGCCTGCCACCTTAC, Rev: GGGAGTCATGGCACCCTGAG; mouse, Dir: CCCTTGTACCCTTCACCAATGAC, Rev: TCACGGTAGATACAATATTTTGAAGCTG and total, Dir: TGCACAGGAGCCAAGAGTGAA, Rev: CACATCACAGCTCCCCACCA. Real time-PCR primers for human BCL2 were: Dir, CTTTGAGTTCGGTGGGGTCA, Rev: GGGCCGTACAGTTCCACAAA (kindly provided by Dr. Lorenzo Montanaro, Bologna, Italy). Expression level was calculated as the difference between Ct of the reference gene (TBP) and Ct of BCL2. In situ detection of HER2 isoform RNA expression was performed using the Basescope assay on FFPE tumor tissues in accordance with guidelines provided by the supplier (Advanced Cell Diagnostics-ACD, Newark, CA, USA). Briefly, 5 μm tissue sections were deparaffinized with

xylene and 100% ethanol. Then slides were pretreated with Hydrogen Peroxide, Target Retrieval solution and Protease III (Pretreatment Reagents Kit, cat. No. 322380, ACD). Slides were then hybridized in HybEZ Hybridization System with probes detecting HER2 TOT (BaseScope Hs-ERBB2-E1ingE18, Cat No. 701131), HER2 FL (BaseScope Hs-ERBB2-E15E16, Cat No. 701121, ACD) and D16 (BaseScope Hs-ERBB2-E15E17, Cat No. 701111). Positive control probe of RNA quality (Hs-PPIB-1zz, Cat No. 701041, ACD) and negative control probe for aspecific signal (DapB-1zz, Cat No. 701021) were also used. After hybridizations, slides were subjected to signal amplification using Basescope Detection Reagent Kit-RED (Cat.No. 322900). After counterstaining with Gill's hematoxylin, tissue sections were examined under a standard bright field microscope at 40× magnification. Expression score was based on the estimated number of dots per cell according to scoring guidelines provided by ACD (scores 0-4, with 4 corresponding to >6 dots/cell).

Western blot

Proteins were extracted from snap-frozen tumor samples in PhosphoSafe Extraction Reagent (Novagen) supplemented with protease inhibitors (Protease Inhibitor Cocktail, Sigma) using gentle MACS Octo Dissociator (Miltenyi Biotech GmbH, Germany). After quantification with DC Protein Assay (Bio-Rad Laboratories, USA), proteins were separated on 8% polyacrylamide gel and then transferred to polyvinylidene difluoride membranes (Bio-Rad Laboratories). After blocking, membranes were incubated with the following primary antibodies: anti-ErbB2 mouse monoclonal antibody (3B5) (Calbiochem Millipore, RRID:AB_213313), anti-phospho-HER2 (Tyr 1248)-R rabbit polyclonal antibody (Santa Cruz Biotechnology, RRID:AB_653115), anti IGF-1Rβ (C-20) rabbit polyclonal antibody (Santa Cruz Biotechnology, RRID:AB_671792), anti-AKT rabbit

polyclonal antibody (RRID:AB_329827), anti-phospho-AKT (Ser473) (D9E) XP rabbit monoclonal antibody (RRID:AB_2315049), anti-p44/42 MAPK (Erk1/2) (137F5) rabbit monoclonal antibody (RRID:AB_390779), anti-phospho-p44/42 MAPK (Erk1/2) (Thr202/Tyr204) rabbit polyclonal antibody (RRID:AB_331646), anti-Stat3 (124H6) mouse monoclonal antibody (RRID:AB_331757), anti-phospho-Stat3(Tyr705) (D3A7) XP rabbit monoclonal antibody (RRID:AB_2491009), anti-EGF R (D38B1) XP rabbit monoclonal antibody (RRID:AB_2246311), anti-phospho-EGF R (Tyr1068) (D7A5) XP rabbit monoclonal antibody (RRID:AB_2096270), anti-HER3/ErbB3 (D22C5)XP rabbit monoclonal antibody (RRID:AB_2721919), anti-phospho- HER3/ErbB3 (Tyr1289) (21D3) rabbit monoclonal antibody (RRID:AB_2099709), anti-HER4/ErbB4 (111B2) rabbit monoclonal antibody (RRID:AB_2099883), anti-phospho- HER4/ErbB4 (Tyr1284) (21A9) rabbit monoclonal antibody (RRID:AB_2099987) (all the twelve former from Cell Signaling Technology) and anti-actin rabbit polyclonal antibody (Sigma-Aldrich, RRID:AB_476693). Protein presence was detected through incubation with the appropriate horseradish peroxidase-labeled secondary antibodies: goat anti-rabbit IgG-HRP (Santa Cruz Biotechnology, RRID:AB_631746 or BIO-RAD, RRID:AB_11125142), goat anti-mouse IgG-HRP (Santa Cruz Biotechnology RRID:AB_631736), followed by chemiluminescent reaction (Clarity Western ECL Substrate, BIO-RAD) before film exposure. Western blot analyses reported in Figure 2 are representative of at least two similar experiments.

Molecular metastasis detection

Lungs, brain and femoral bone marrow were collected from xenograft-bearing mice in cold PBS. Ovaries were frozen directly in vials, in liquid nitrogen, for nucleic acids extraction. Lungs and brain were minced with scissors, bone marrow was flushed from both femurs in PBS, then tissue suspensions were

passed through a 70 μm cell strainer (Becton Dickinson, Bedford, MA, USA) to obtain homogeneous cell suspensions. Known aliquots (1/16 of the brain, 1/6 of the lung and ¼ of each femoral bone marrow) were used to obtain cellular pellets of each organ for quantitative comparison. Genomic DNA was extracted with 10 mM Tris-HCl buffer pH 8.3 containing 50 mM KCl, 2.5 mM MgCl2, 0.01% gelatin, 0.45% Igepal, 0.45% Tween 20 and 120 mg/ml proteinase K (all reagents from Sigma, Milan, Italy) by overnight incubation at 56°C followed by 30 min incubation at 95°C to inactivate the proteinase K. A sequence of the α-satellite region of the human chromosome 17 was amplified. Primer and probe sequences were derived from Becker et al.[6] with the sole alteration that the probe carried the non-fluorescent quencher dye TAMRA at the 39-end[7]. 1 ng DNA aliquot per sample was amplified using 250 nM primers and 100 nM probe in a final volume of 25 μl of Sso Advanced Universal Probes Supermix (Bio-Rad, Italy). Real Time-PCR was performed using a Thermal Cycler CFX96 real time system C1000 (Bio-Rad). DNA extracted from mouse tissues showed no amplification up to 40 cycles. To quantify human cells, a standard curve was constructed by adding scalar amounts of MDA-MB-453 human cells to a constant number of mouse cells. Ct (threshold cycle) values obtained from the experimental samples were interpolated in the standard curve run in each PCR (Bio-Rad CFX Manager)[7]. The final number of disseminated tumor cells per organ was obtained considering the fraction analyzed for each organ. In some case half of the lung and brain, one ovary, one femoral bone and the liver were fixed in 10% neutral buffered formalin solution for histological examination.

Supplementary references

1. Coates, A. S. *et al.* Tailoring therapies--improving the management of early breast cancer: St Gallen International Expert Consensus on the

Primary Therapy of Early Breast Cancer 2015. *Ann.Oncol.* **26**, 1533–1546 (2015).

2. Wolff, A. C. *et al.* Recommendations for human epidermal growth factor receptor 2 testing in breast cancer: American Society of Clinical Oncology/College of American Pathologists clinical practice guideline update. *J.Clin Oncol.* **31**, 3997–4013 (2013).
3. Mitra, D. *et al.* An oncogenic isoform of HER2 associated with locally disseminated breast cancer and trastuzumab resistance. *Mol.Cancer Ther.* **8**, 2152–2162 (2009).
4. Palladini, A. *et al.* HER2 isoforms co-expression differently tunes mammary tumor phenotypes affecting onset, vasculature and therapeutic response. *Oncotarget* **8**, 54444–54458 (2017).
5. Bieche, I. *et al.* Vasculature analysis of patient derived tumor xenografts using species-specific PCR assays: evidence of tumor endothelial cells and atypical VEGFA-VEGFR1/2 signalings. *BMC.Cancer* **14**, 178 (2014).
6. Becker, M. *et al.* Sensitive PCR method for the detection and real-time quantification of human cells in xenotransplantation systems. *Br.J.Cancer* **87**, 1328–1335 (2002).
7. Nanni, P. *et al.* Multiorgan metastasis of human HER-2+ breast cancer in Rag2-/-;Il2rg-/- mice and treatment with PI3K inhibitor. *PLoS One* **7**, e39626 (2012).

Supplementary Table S1. Clinical features of implanted primary tumors

Parameter	Type	Number of cases	%[a]
Histology	Invasive carcinoma of no special type (NST)	54	88.5
	Invasive lobular carcinoma	6	9.8
	Invasive medullary carcinoma	1	1.6
Grade	III	30	49.2
	II	21	34.4
	I	10	16.4
Tumor size	T1	32	52.5
	T2	27	44.3
	>T2	2	3.3
Node invasion	N0	26	42.6
	N1	17	27.9
	≥N2	13	21.3
	Not evaluated	5	8.2
Hormone receptors	ER+ and/or PR+	50	82.0
	ER- and PR-	11	18.0
HER2 expression	HER2 overexpression or amplification[b]	14	23.0
Proliferation	Ki-67 high	26	42.6
	Ki-67 intermediate	13	21.3
	Ki-67 low	22	36.1
Subtypes	Luminal A	28	45.9
	Luminal B	13	21.3
	HER2 triple-positive	9	14.8
	HER2-positive	5	8.2
	Triple-negative/basal like	6	9.8

[a]over total number of cases studied (n=61)

[b]FISH analysis

Supplementary Table S2. Stability of phenotypic features along in vivo passaging

PDX	Subtype of the original tumor	Host	In vivo passage[a]	Hormone receptors: ER % (median)	Hormone receptors: PR % (median)	Ki-67	BCL2	p53 % (median)	EGFR/HER1	HER2 Score	HER2 FISH
BRS7	Luminal B with mixed phenotype	**Patient** (Histology 1)		**100**	**35**	**Low**	**Pos**	**80**	**Neg**	**0**	**n.d.**
		**Patient** (Histology 2)		**0**	**0**	**High**	**Neg**	**45**	**Pos**	**0**	**n.d.**
		Mice	1 (2)	7	0.1	High	Neg	72	Pos	0/1	n.d.
			2 (3)	5	0.2	High	Neg	74	Pos	0/1	n.d.
			3 (5)	2	0	High	Neg	91	Pos	0	n.d.
			4 (5)	1	0	High	Neg	95	Pos	0/1	n.d.
			5 (3)	0,5	0	High	Neg	90	Pos	0/1	n.d.
BRS18	Luminal B	**Patient**		**100**	**0.4**	**High**	**Int**	**6**	**Neg**	**2**	**neg**
		Mice	1 (1)	100	0	High	Int	0.1	Neg	0/1	n.d.
			2 (2)	100	0.1	High	Int	0.6	Neg	1/2	n.d.
			3 (3)	100	0.1	High	Int/Pos	15	Neg	1/2	n.d.
			4 (1)	100	0.2	High	Int	28	Neg	1/2	n.d.
			5 (3)	100	0	High	Int	20	Neg	1/2	n.d.
			6 (3)	100	0.2	High	Int/Pos	18	Neg	1/2	n.d.
			7 (4)	100	0.2	High	Int	19	Neg	1/2	n.d.
			8 (4)	100	0,1	High-Int	Int/Pos	17	Neg	1/2	n.d.
			9 (5)	100	0.1	High	Int/Pos	16	Neg	1/2	n.d.
			10 (1)	100	0	Int	Int/Pos	13	Neg	1	n.d.
BRB15	HER2-Triple positive	**Patient**		**100**	**60**	**Int**	**Pos**	**90**	**Neg**	**2**	**ampl**
		Mice	1 (1)	pos	pos	High	Pos	90	Neg	1	neg
		Mice	2 (2)	100	70	Int	Pos	70	Neg	0	n.d.
BRS45	HER2-positive	**Patient**		**0**	**0**	**High**	**Neg**	**0**	**Pos**	**3**	**n.d.**
		Mice	1 (1)	0	0	High	Neg	0	Neg	3	n.d.
			2 (2)	0	0	High	Neg	0	Int/pos	3	n.d.
			3 (4)	0	0	High	Neg	0	Int/pos	3	n.d.
			4 (5)	0	0	High	Neg	0	Int/pos	3	n.d.
			5 (6)	0	0	High	Neg	0	Int/pos	3	n.d.
			6 (1)	0	0	High	Neg	0	neg/Int	3	n.d.

			7 (2)	0	0	High	Neg	0	Int	3	n.d.
BRB4	HER2-positive	**Patient**		**0**	**0**	**High**	**Pos**	**100**	**Neg**	**2**	**ampl**
		Mice	2 (1)	0	0	n.d.	Pos	98	Neg	3	ampl
			3 (1)	0	0	High	Pos	100	Neg	3	ampl
			4 (1)	0	0	High	Pos	100	Neg	3	ampl
			5 (1)	0	1	High	Pos	100	Neg	3	ampl
			6 (1)	0	3	High	Pos	100	Neg	3	ampl
			7 (1)*	0	2	High	Pos	100	Neg	3	ampl
			11 (3)	0	0	high	Pos	100	Neg	3	ampl
			12 (1)*	0	0	High	Pos/Int	100	Neg	3	ampl
			14 (5)*	0	0	High	Pos/Int	100	Neg	3	ampl
			15 (1)*	0	0	High	Pos/Int	100	Neg	3	ampl
			16 (1)	0	0	High	Low	100	Neg	3	n.d.
			17 (1)*	0	0	High	Pos/Int	100	Neg	3	n.d.
			22 (2)*	0	0	High	Low	100	Neg	3	n.d.
			23 (4)*	0	0	High	Low/Int	100	Neg	3	n.d.
			26 (1)*	0	0	High	Neg	100	Neg	3	n.d.
BRS51	Triple negative	**Patient**		**0**	**0**	**High**	**Neg**	**0**	**Pos**	**0**	**n.d.**
		Mice	1 (1)	0	0	High	Int	0	Pos	0	n.d.
			2 (2)	0	0	High	Neg/Int	0	Pos	0/1	n.d.
			3 (2)	0	0	High	Neg/Int	0	Pos	0	n.d.
			4 (4)	0	0	High	Neg/Int	0	Pos/Int	0	n.d.
			5 (1)	0	0	High	Int	0	Pos/Int	0	n.d.
			6 (3)	0	0	High	Int/Pos	0	Pos	0	n.d.

[a]number of mice is reported in parentheses

*A1 subline

Abbreviations: ampl= amplified; Int=intermediate; n.d.=not determined; Neg=negative; Pos= positive.

Supplementary Figure S1. In situ hybridization of HER2 full-length and HER2-D16 mRNA isoforms in HER2-amplified PDX-BRB4 (BaseScope assay on FFPE tissue sections). (A) HER2 probe hybridizing all isoforms, score 4 (>6 dots/cell, >10% positive cells have dot clusters); (B) HER2 probe hybridizing HER2 full-length isoform, score 4; (C) D16 probe hybridizing HER2-D16 splice variant, score 2 (2-3 dots/cell); (D) negative control probes. Magnification 40×, Scale bar = 33 μm.

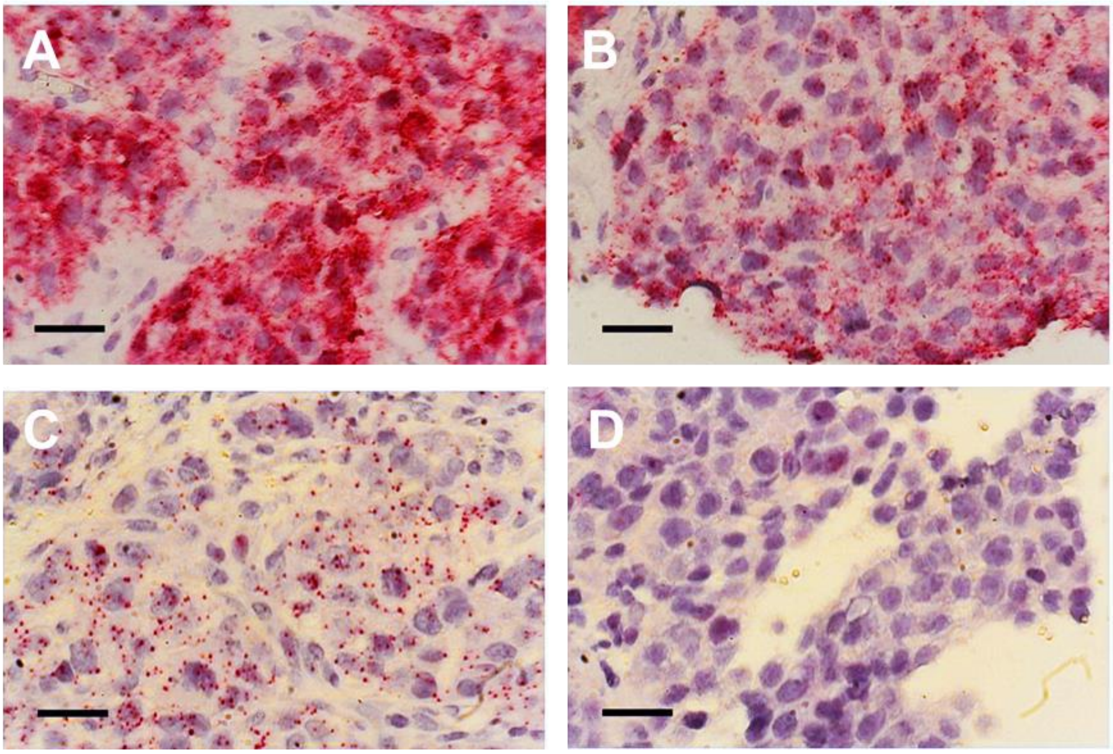

Supplementary Figure S2. Expression of HER2 full-length (open black circles) and HER2-D16 (red closed squares) mRNA isoforms in HER2-positive PDX after serial in vivo passage. Results from RT-PCR analysis are expressed as ΔCt = Ct of relevant gene – Ct of hTBP reference gene. Individual samples are plotted, with median (horizontal line). A negative value means that relevant gene is more expressed than reference gene, while a positive value means that relevant gene is less expressed than reference gene. RT-PCR analysis was performed in parallel on a high HER2-positive (BT474) and a HER2-negative (RH4) human tumor cell lines. Mean ΔCt of BT474 positive controls were: HER2 = -6.97; D16 = -2.75. Mean ΔCt of RH4 negative controls were: HER2 = 4.74; D16 = 9.11.

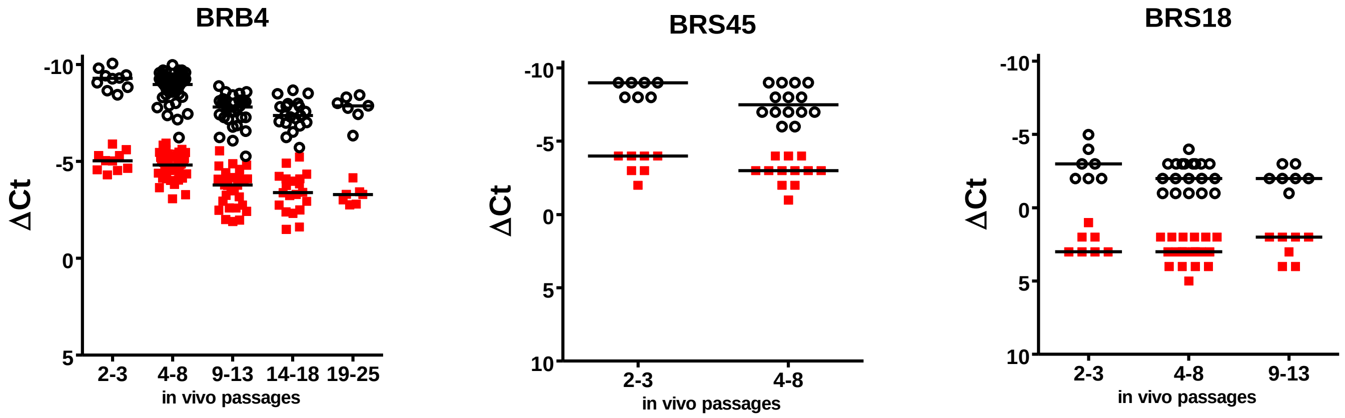

Supplementary Figure S3. Effect of in vitro treatment with neratinib on signaling of PDX-BRB4 and PDX-BRS18. Primary cell cultures derived from PDX-BRB4 (in vivo passage 9) and from PDX-BRS18 (in vivo passage 7) were treated for 24 h with neratinib (40 nM). Controls were treated with vehicle alone (dimethyl sulfoxide). Full-length blots are presented in Supplementary Figure S8 B.

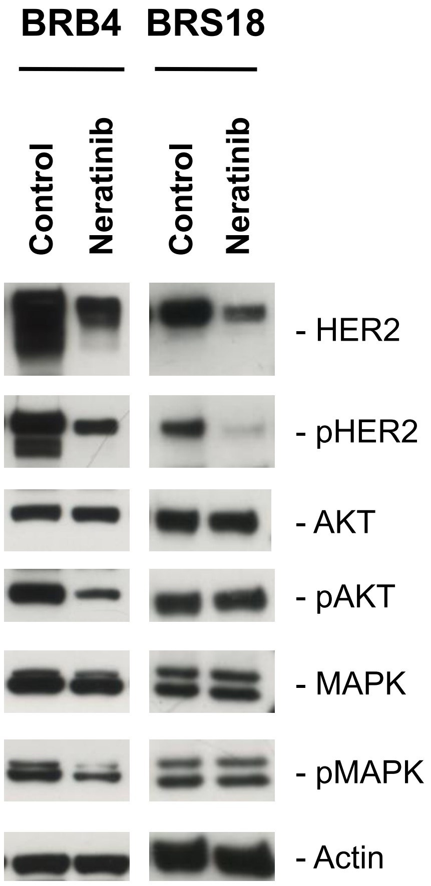

Supplementary Figure S4. β-galactosidase activity in PDX-BRB4. Cell staining was performed on primary cell cultures derived from PDX-BRB4 subline A1 at passage 9 (left) and 28 (right) after 25 days of culture (magnification ×100).

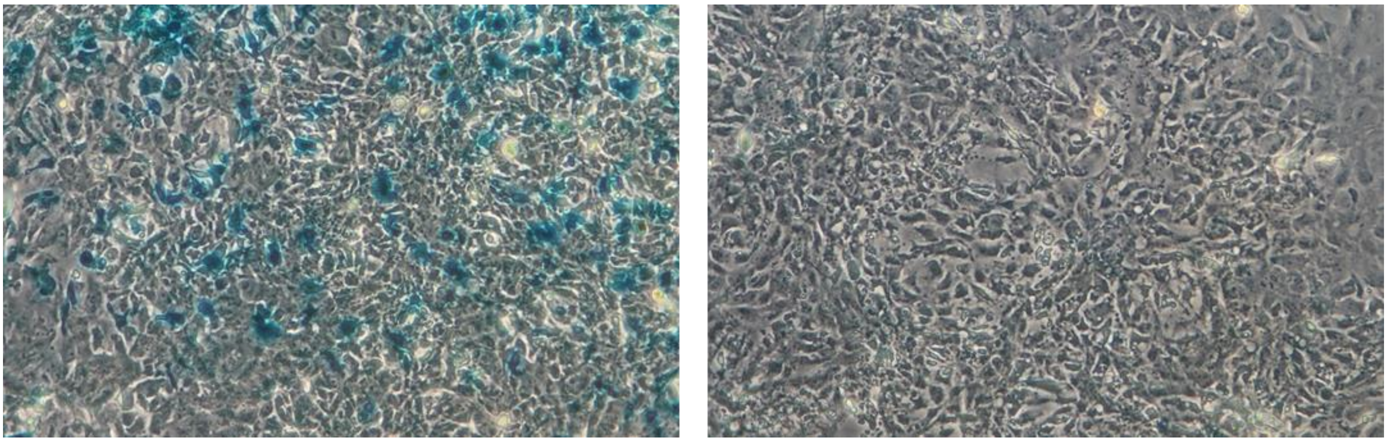

Supplementary Figure S5. Principal component analysis of the RNAseq experiments on PDX-BRB4 sublines. Symbols: green, A1 subline at in vivo passage 4; red, A1 subline at passage 17 (progressed); black, A1 subline at passage 24 (progressed); purple, C1 subline at passage 4; blue, C1 subline at passage 17.

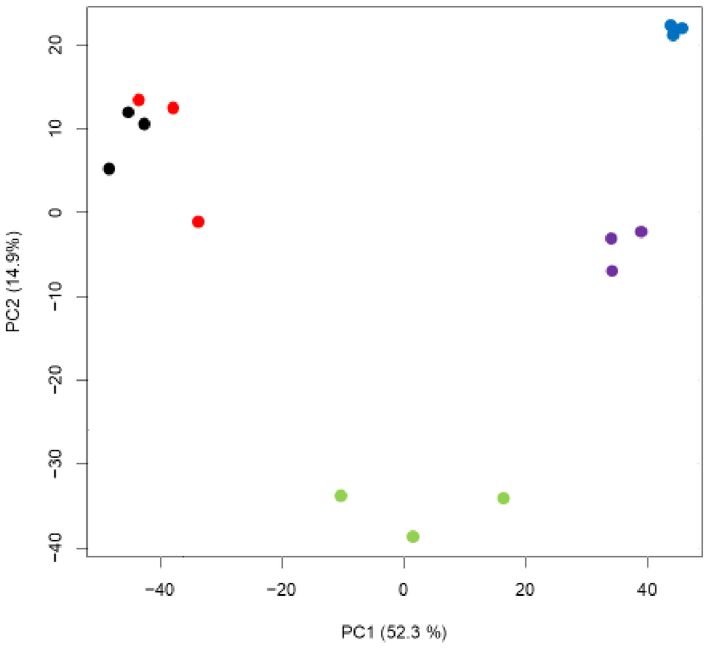

Supplementary Figure S6. IPA network for the 481 DE (194 UP and 287 DW) detected by hierarchical clustering. Genes were connected using only direct interactions.

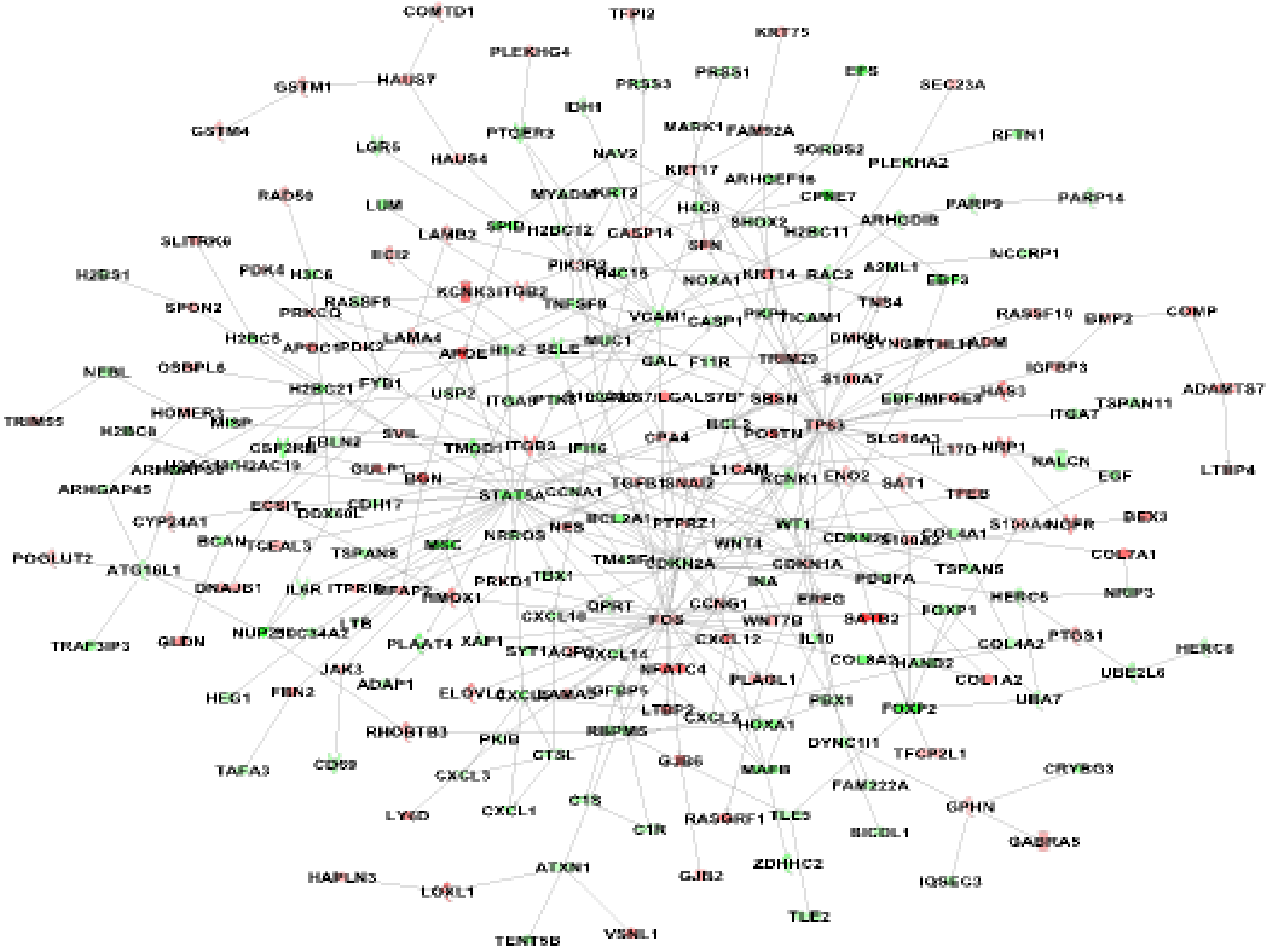

Supplementary Figure S7. HER-2 expression of trastuzumab-sensitive and trastuzumab-resistant PDX-BRB4 tumors. Surface HER-2 expression was determined by flow cytometry (primary antibody: mouse anti-human HER2, clone MGR2, Alexis Biochemical, Enzo life Sciences; secondary antibody: Anti-mouse IgG AF488, Thermo Fisher Scientific). Each bar represents the mean + SEM of eight different tumors, the two samples were not significantly different by the Student's *t* test (p=0.11).

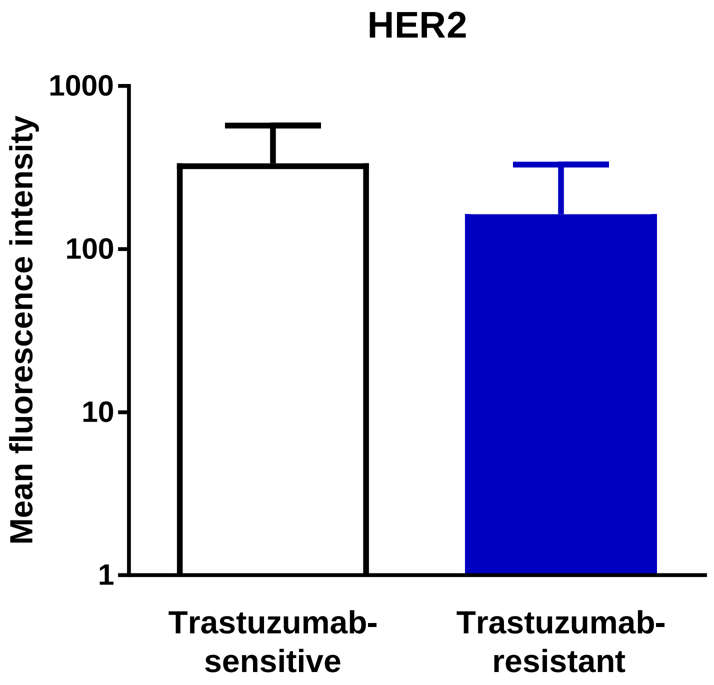

Supplementary Figure S8. Full-length blots. (A) Full length blots of Figure 2 (panel G-I) showing the effect of indicated treatments on signaling of PDX-BRB4, PDX-BRS45 and PDX-BRS18. PDX-BRS18 blots reporting lanes "Tamoxifen, Tam+Neratinib and Contol 2" were mirrored in Figure 2 (panel I) to improve the clarity of the data presentation. (B) Full-length blots of Supplementary Figure S3. (C) MagicMark XP Western Protein Standard (Thermo Fisher Scientific).

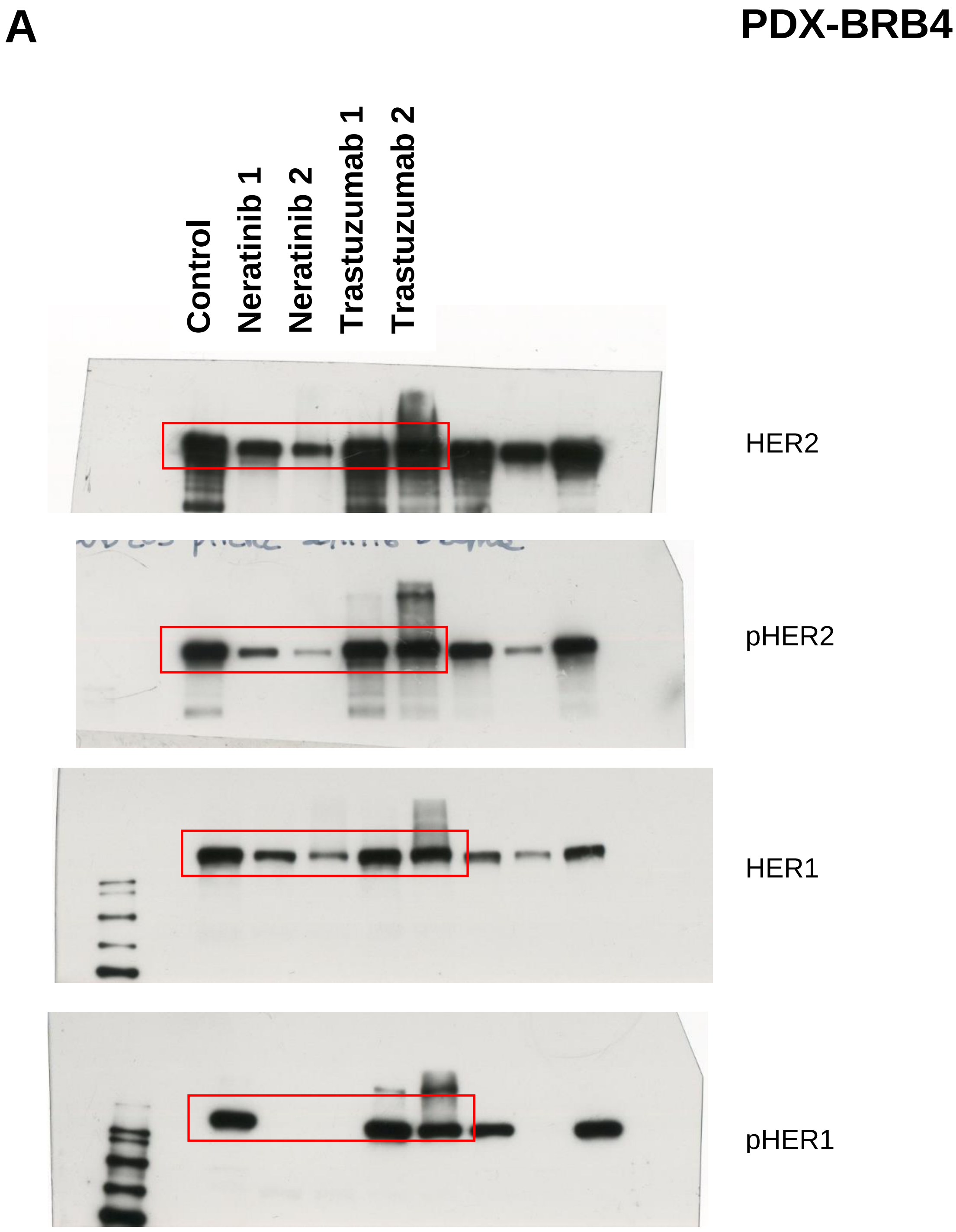

Supplementary Figure S8 continued

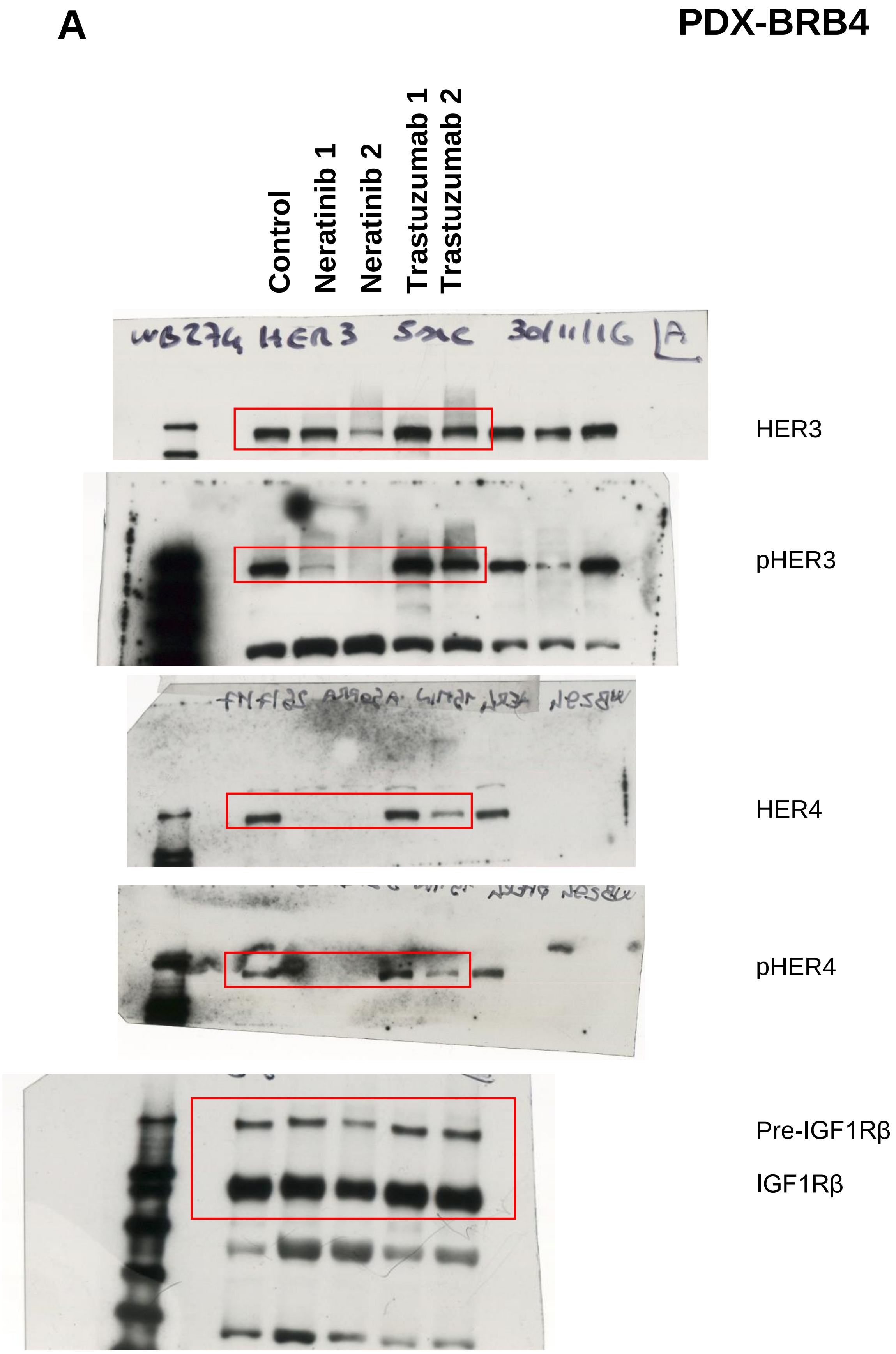

Supplementary Figure S8 continued

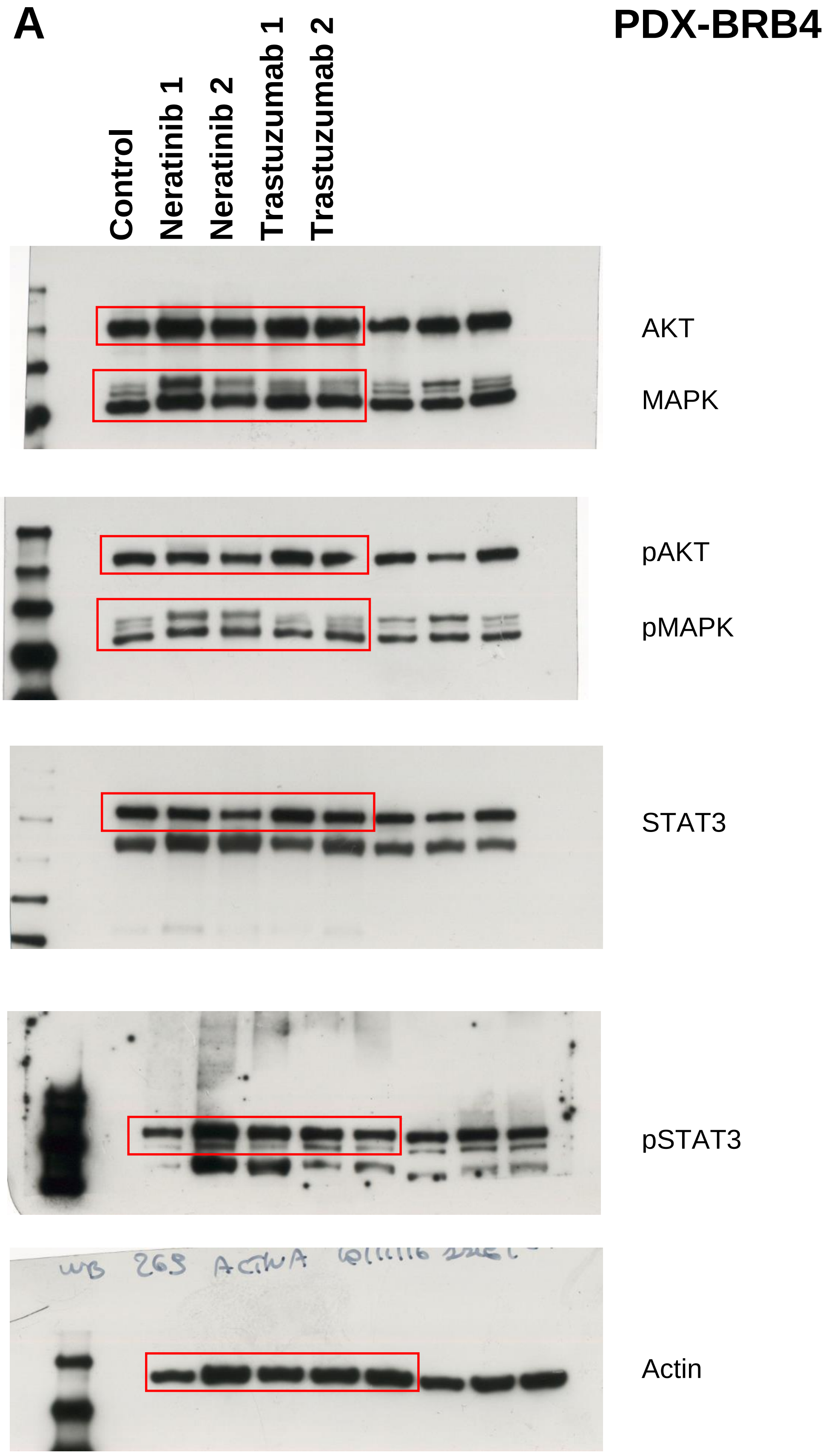

Supplementary Figure S8 continued

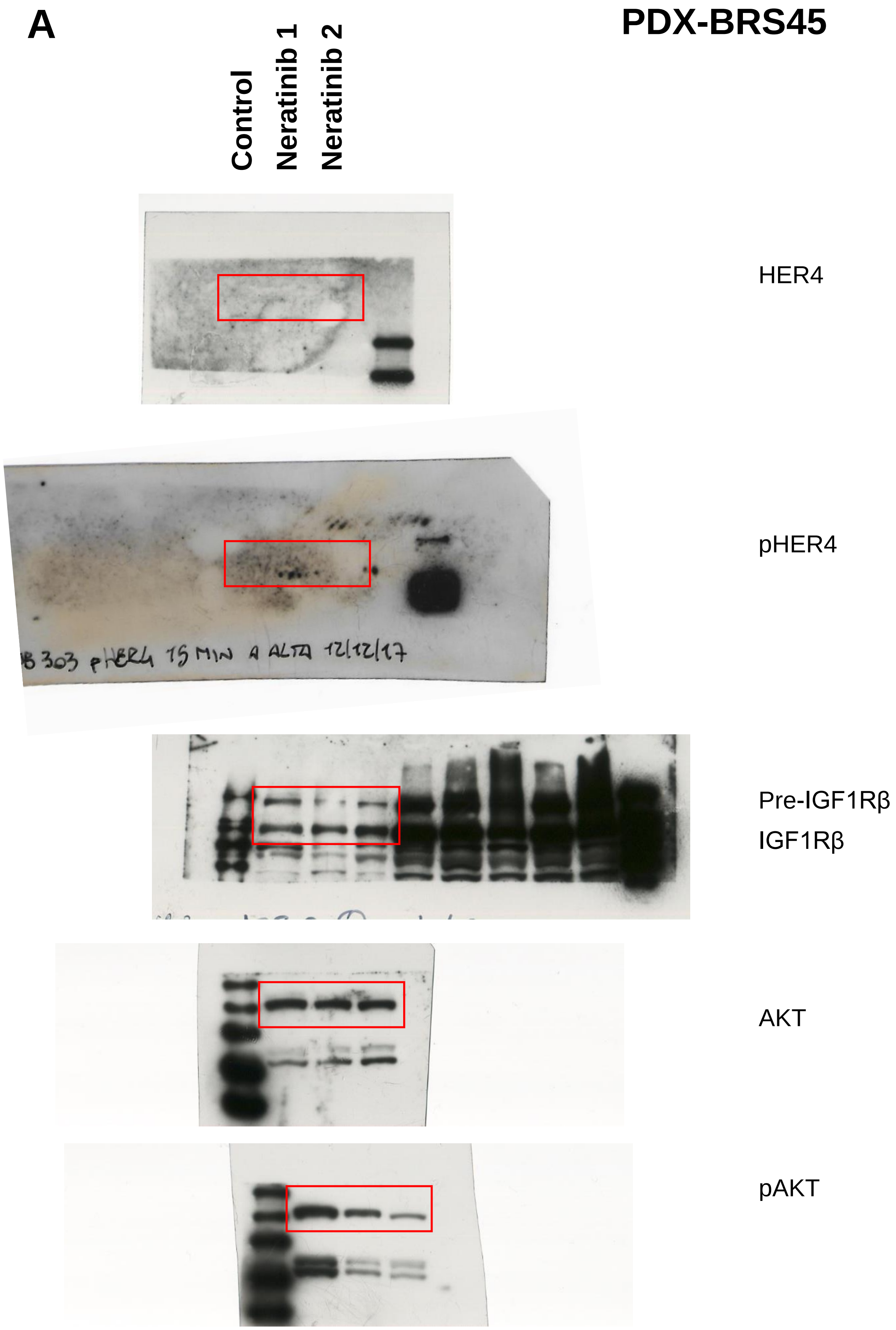

Supplementary Figure S8 continued

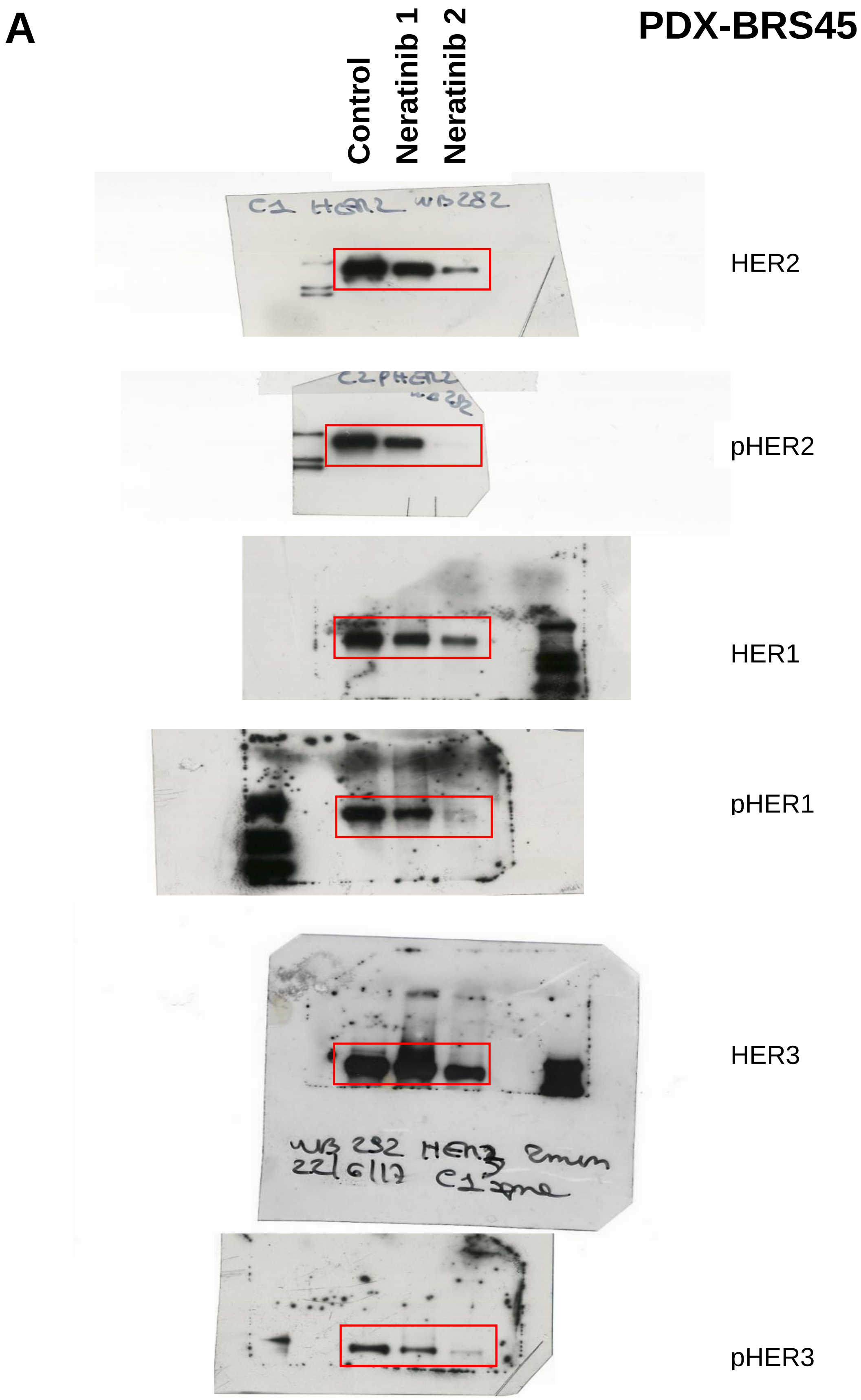

Supplementary Figure S8 continued

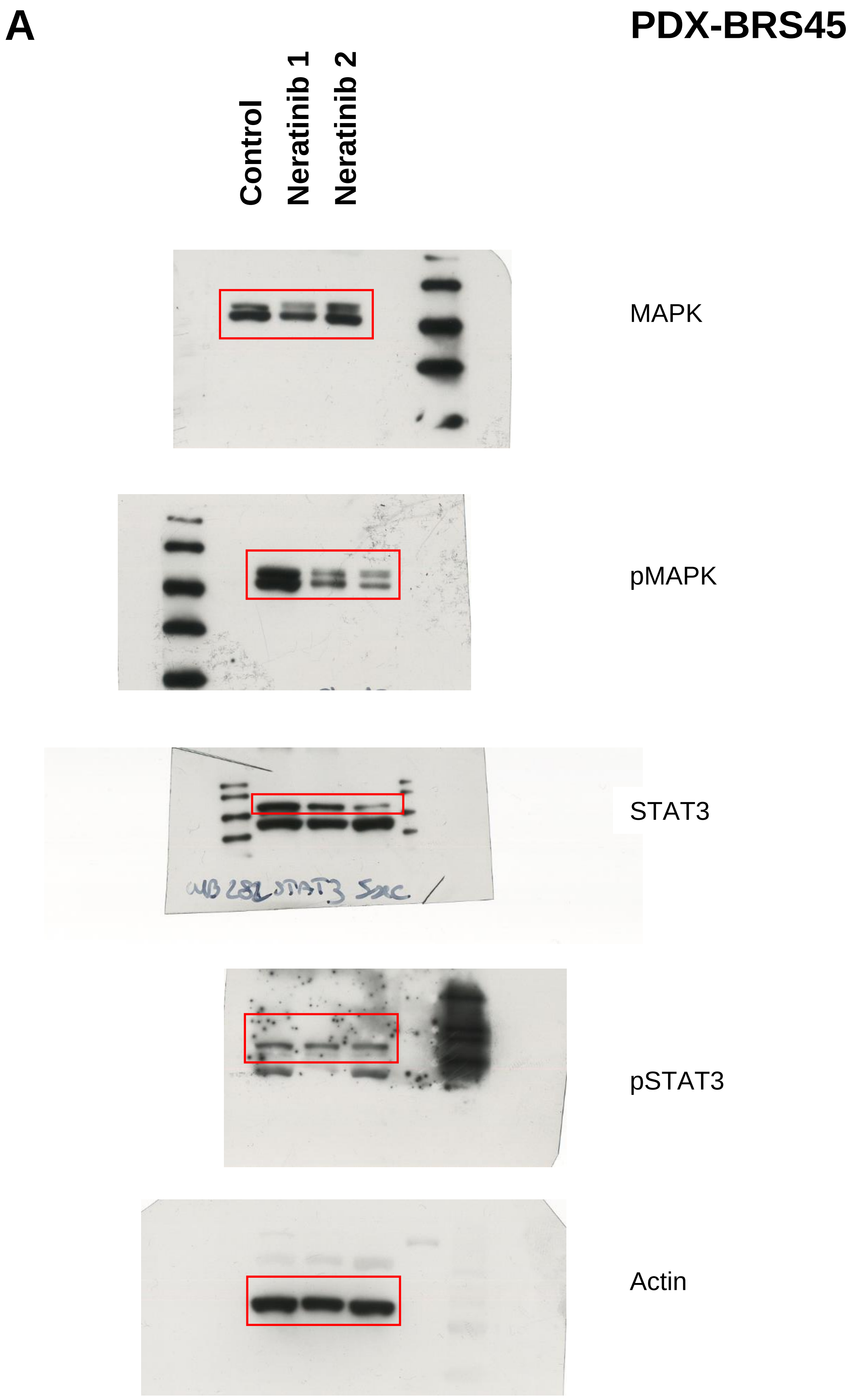

Supplementary Figure S8 continued

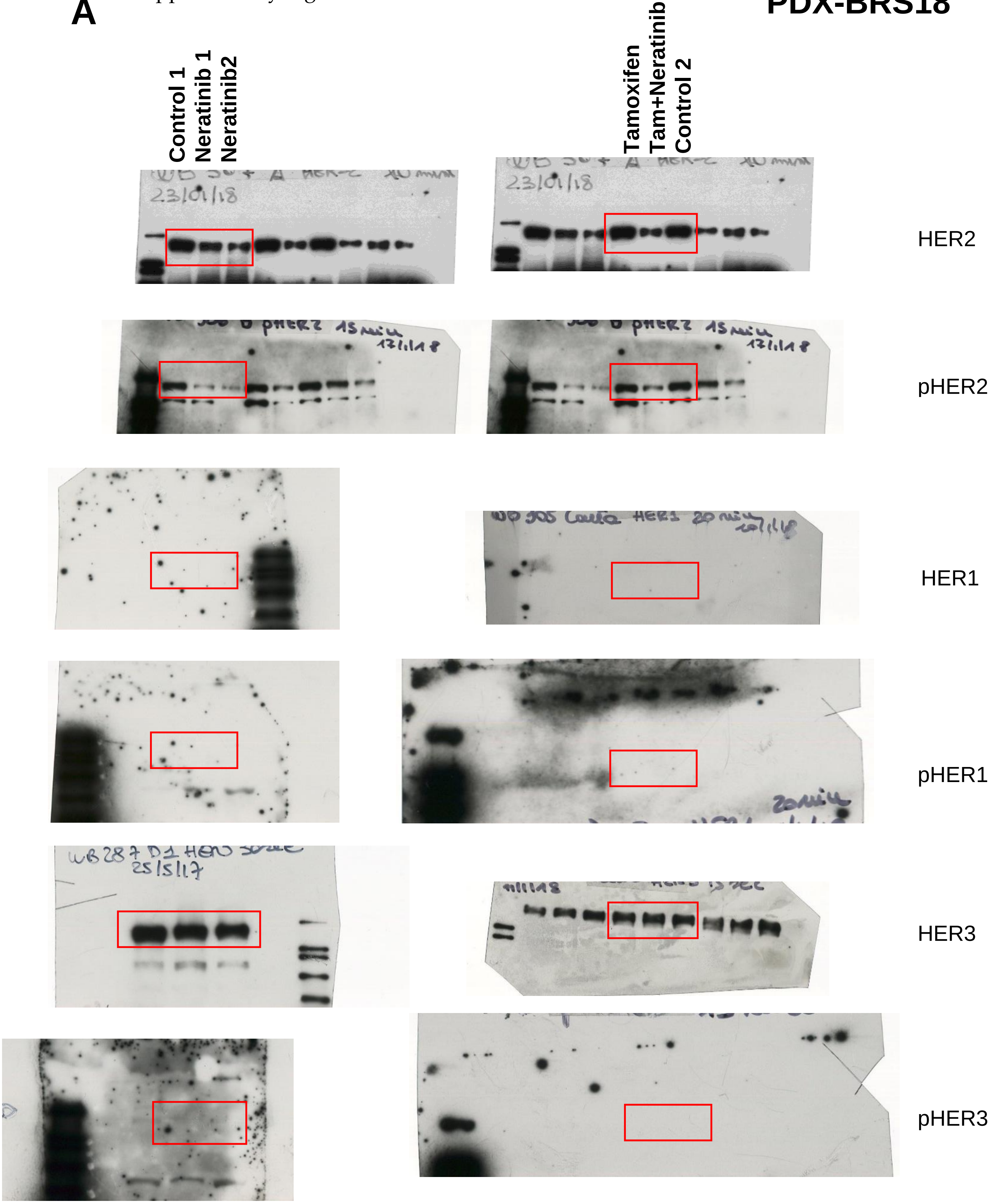

Supplementary Figure S8 continued

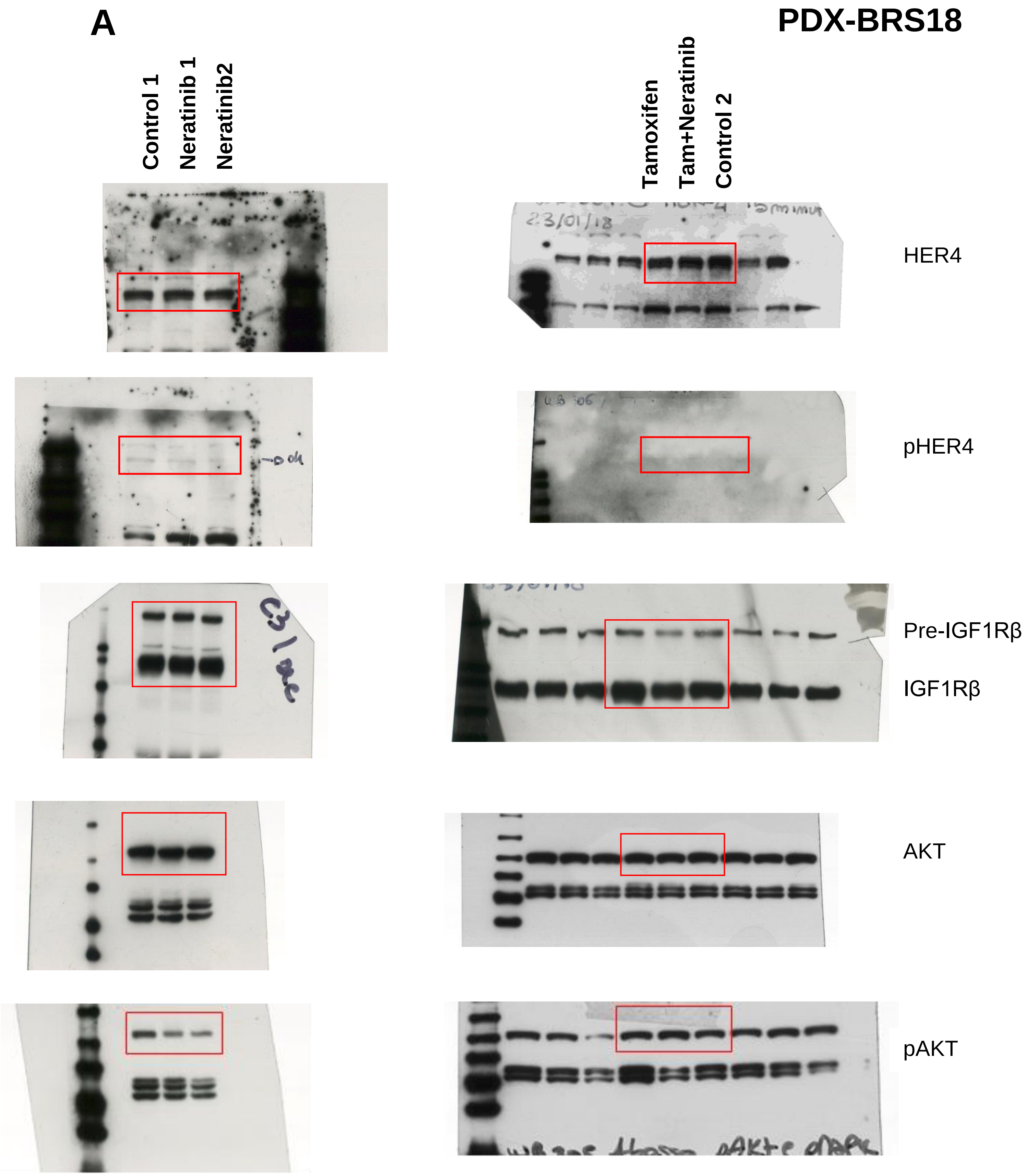

Supplementary Figure S8 continued

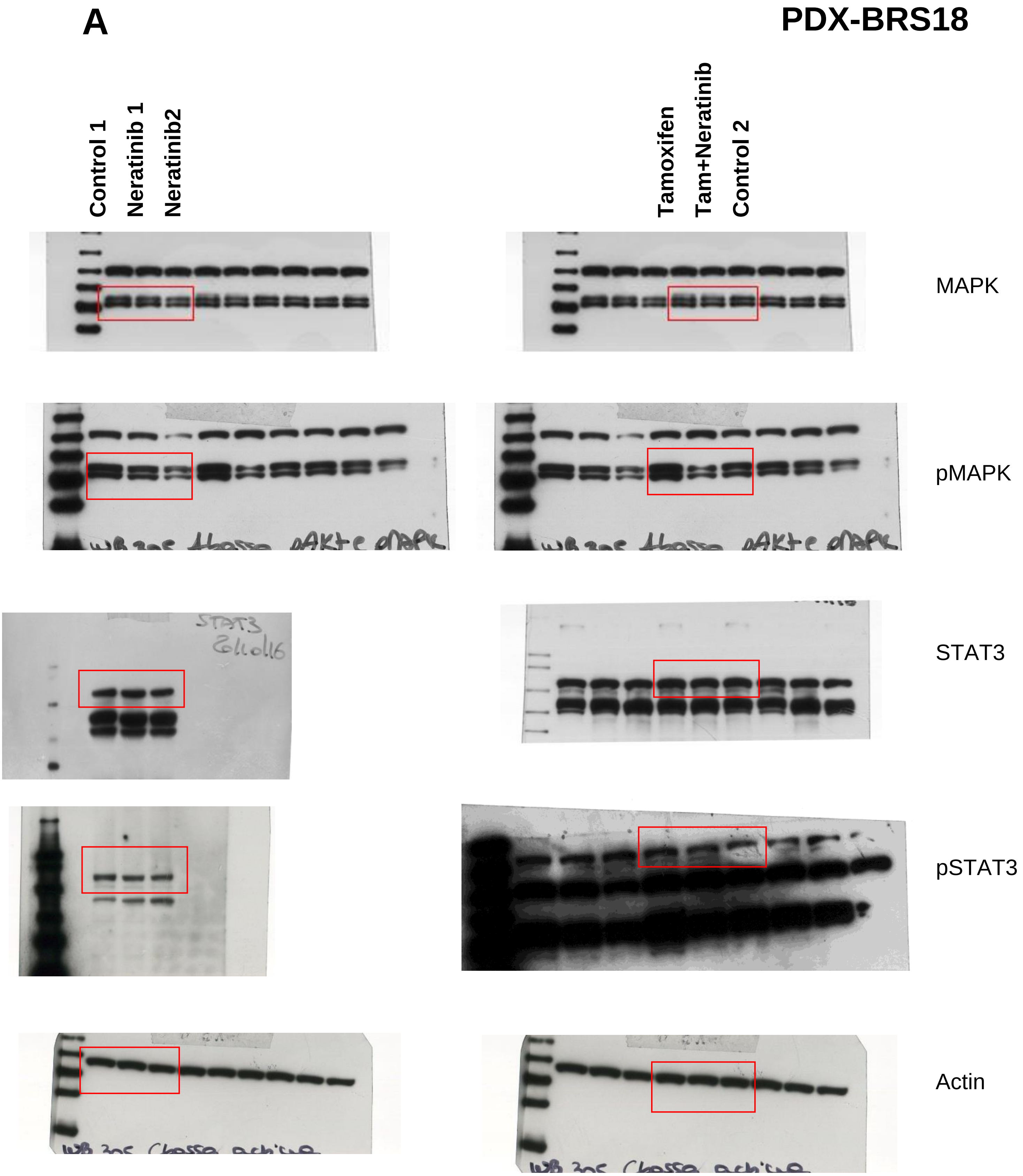

Supplementary Figure S8 continued

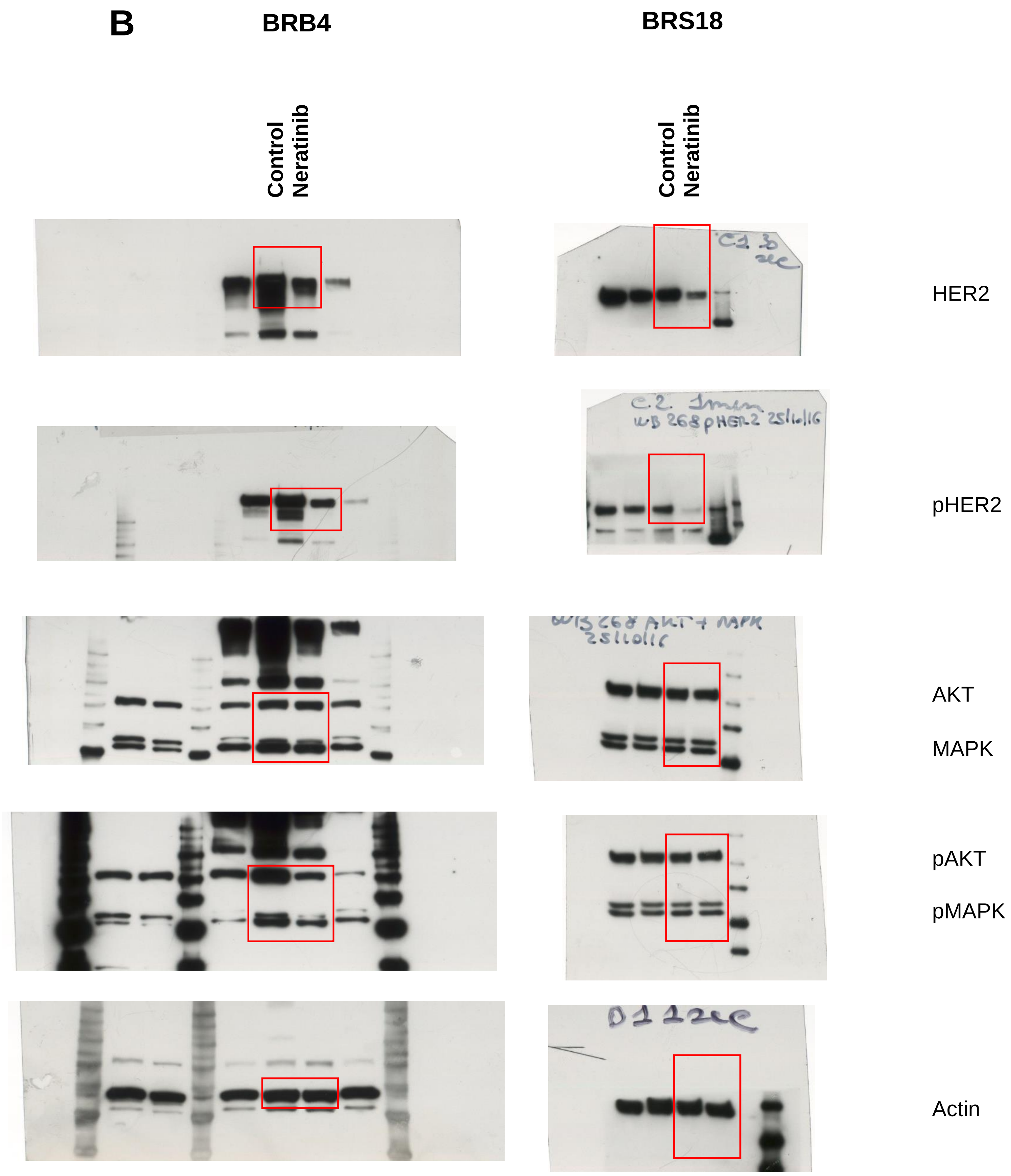

Supplementary Figure S8 continued

C

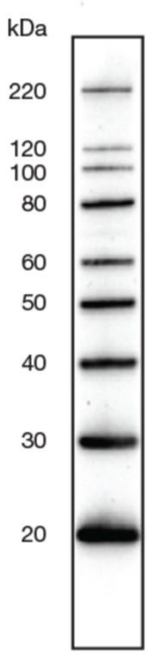

Supplement: Supplementary file 2 — Supplementary Information 2. [file 41598_2021_81085_MOESM2_ESM.pdf]
